# Supplementary figures and images for: Hybrid endosomal coats contain different classes of sorting nexins (part 1 of 2)
Source: EMBO J. 2026 Feb 16;45(7):2278–305. doi: 10.1038/s44318-026-00716-0 (PMC13043683; doi:10.1038/s44318-026-00716-0)

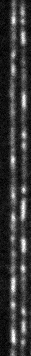

Supplement: Supplementary file 2 — Source data Fig. 1 [file 44318_2026_716_MOESM2_ESM.zip › SD Figure 1/Ear1-547.tif]

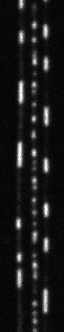

Supplement: Supplementary file 2 — Source data Fig. 1 [file 44318_2026_716_MOESM2_ESM.zip › SD Figure 1/Ear1_Retromer-mClover.tif]

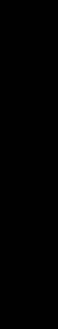

Supplement: Supplementary file 2 — Source data Fig. 1 [file 44318_2026_716_MOESM2_ESM.zip › SD Figure 1/NoCargo_Retromer-mClover.tif]

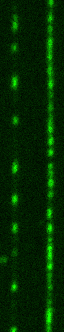

Supplement: Supplementary file 2 — Source data Fig. 1 [file 44318_2026_716_MOESM2_ESM.zip › SD Figure 1/Ste13_Retromer-mClover.tif]

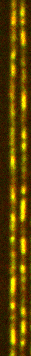

Supplement: Supplementary file 2 — Source data Fig. 1 [file 44318_2026_716_MOESM2_ESM.zip › SD Figure 1/Retromer-mClover_Ear1-547_merged.tif]

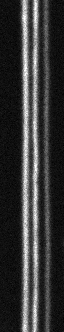

Supplement: Supplementary file 2 — Source data Fig. 1 [file 44318_2026_716_MOESM2_ESM.zip › SD Figure 1/Vps10_Cy5_5-PE.tif]

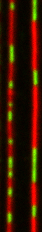

Supplement: Supplementary file 2 — Source data Fig. 1 [file 44318_2026_716_MOESM2_ESM.zip › SD Figure 1/SNX3_merged.tif]

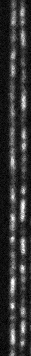

Supplement: Supplementary file 2 — Source data Fig. 1 [file 44318_2026_716_MOESM2_ESM.zip › SD Figure 1/Retromer-mClover.tif]

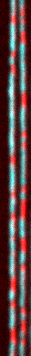

Supplement: Supplementary file 2 — Source data Fig. 1 [file 44318_2026_716_MOESM2_ESM.zip › SD Figure 1/Ear1-547_Cy5-5_merged.tif]

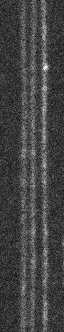

Supplement: Supplementary file 2 — Source data Fig. 1 [file 44318_2026_716_MOESM2_ESM.zip › SD Figure 1/Vps10_Retromer-mClover.tif]

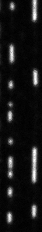

Supplement: Supplementary file 2 — Source data Fig. 1 [file 44318_2026_716_MOESM2_ESM.zip › SD Figure 1/SNX3_Retromer-mClover.tif]

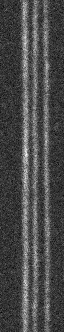

Supplement: Supplementary file 2 — Source data Fig. 1 [file 44318_2026_716_MOESM2_ESM.zip › SD Figure 1/Vps10_mScarlettI-SNX3.tif]

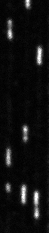

Supplement: Supplementary file 2 — Source data Fig. 1 [file 44318_2026_716_MOESM2_ESM.zip › SD Figure 1/SNX-BAR_Retromer-mClover.tif]

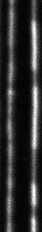

Supplement: Supplementary file 2 — Source data Fig. 1 [file 44318_2026_716_MOESM2_ESM.zip › SD Figure 1/SNX3_TR-DHPE.tif]

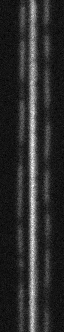

Supplement: Supplementary file 2 — Source data Fig. 1 [file 44318_2026_716_MOESM2_ESM.zip › SD Figure 1/Ear1_Cy5_5-PE.tif]

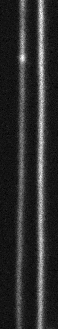

Supplement: Supplementary file 2 — Source data Fig. 1 [file 44318_2026_716_MOESM2_ESM.zip › SD Figure 1/NoCargo_Cy5_5-PE.tif.tif]

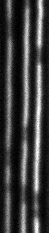

Supplement: Supplementary file 2 — Source data Fig. 1 [file 44318_2026_716_MOESM2_ESM.zip › SD Figure 1/SNX-BAR_TR-DHPE.tif]

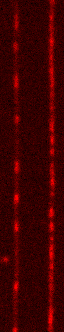

Supplement: Supplementary file 2 — Source data Fig. 1 [file 44318_2026_716_MOESM2_ESM.zip › SD Figure 1/Ste13_mScarlettI-SNX3.tif]

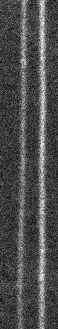

Supplement: Supplementary file 2 — Source data Fig. 1 [file 44318_2026_716_MOESM2_ESM.zip › SD Figure 1/NoCargo_mScarlettI-SNX3.tif]

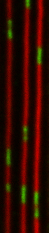

Supplement: Supplementary file 2 — Source data Fig. 1 [file 44318_2026_716_MOESM2_ESM.zip › SD Figure 1/SNX-BAR_merged.tif]

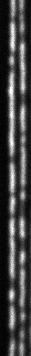

Supplement: Supplementary file 2 — Source data Fig. 1 [file 44318_2026_716_MOESM2_ESM.zip › SD Figure 1/Cy5-5.tif]

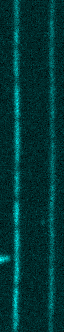

Supplement: Supplementary file 2 — Source data Fig. 1 [file 44318_2026_716_MOESM2_ESM.zip › SD Figure 1/Ste13_Cy5_5-PE.tif]

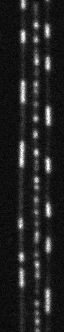

Supplement: Supplementary file 2 — Source data Fig. 1 [file 44318_2026_716_MOESM2_ESM.zip › SD Figure 1/Ear1_mScarlettI-SNX3.tif]

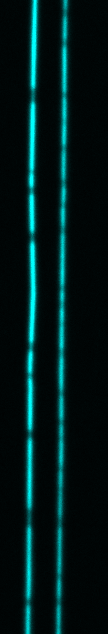

Supplement: Supplementary file 3 — Source data Fig. 2 [file 44318_2026_716_MOESM3_ESM.zip › SD Figure 2/Cy5-5_PE.tif]

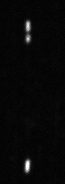

Supplement: Supplementary file 3 — Source data Fig. 2 [file 44318_2026_716_MOESM3_ESM.zip › SD Figure 2/SNX3_25nM_Retromer-mClover_GREEN.tif]

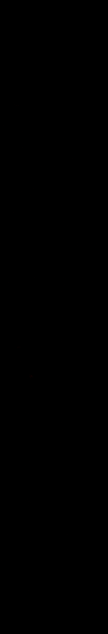

Supplement: Supplementary file 3 — Source data Fig. 2 [file 44318_2026_716_MOESM3_ESM.zip › SD Figure 2/mScarletI-SNX3.tif]

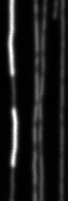

Supplement: Supplementary file 3 — Source data Fig. 2 [file 44318_2026_716_MOESM3_ESM.zip › SD Figure 2/SNX-BAR_100nM-Retromer-mClover_RED.tif]

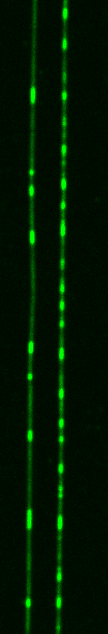

Supplement: Supplementary file 3 — Source data Fig. 2 [file 44318_2026_716_MOESM3_ESM.zip › SD Figure 2/GFP-SNX3.tif]

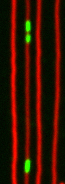

Supplement: Supplementary file 3 — Source data Fig. 2 [file 44318_2026_716_MOESM3_ESM.zip › SD Figure 2/SNX3_25nM_Retromer-mClover_merged.tif]

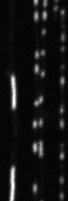

Supplement: Supplementary file 3 — Source data Fig. 2 [file 44318_2026_716_MOESM3_ESM.zip › SD Figure 2/SNX-BAR_100nM-Retromer-mClover_GREEN.tif]

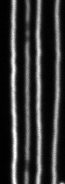

Supplement: Supplementary file 3 — Source data Fig. 2 [file 44318_2026_716_MOESM3_ESM.zip › SD Figure 2/SNX3_25nM_Retromer-mClover_RED.tif]

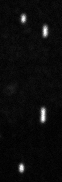

Supplement: Supplementary file 3 — Source data Fig. 2 [file 44318_2026_716_MOESM3_ESM.zip › SD Figure 2/SNX3_100nM_Retromer-mClover_GREEN.tif]

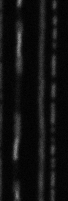

Supplement: Supplementary file 3 — Source data Fig. 2 [file 44318_2026_716_MOESM3_ESM.zip › SD Figure 2/SNX-BAR_25nM-Retromer-mClover_GREEN.tif]

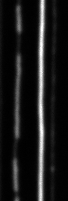

Supplement: Supplementary file 3 — Source data Fig. 2 [file 44318_2026_716_MOESM3_ESM.zip › SD Figure 2/SNX-BAR_25nM-Retromer-mClover_RED.tif]

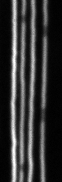

Supplement: Supplementary file 3 — Source data Fig. 2 [file 44318_2026_716_MOESM3_ESM.zip › SD Figure 2/SNX3_100nM_Retromer-mClover_RED.tif]

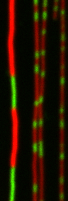

Supplement: Supplementary file 3 — Source data Fig. 2 [file 44318_2026_716_MOESM3_ESM.zip › SD Figure 2/SNX-BAR_100nM-Retromer-mClover_merged.tif]

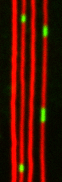

Supplement: Supplementary file 3 — Source data Fig. 2 [file 44318_2026_716_MOESM3_ESM.zip › SD Figure 2/SNX3_100nM_Retromer-mClover_merged.tif]

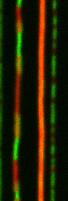

Supplement: Supplementary file 3 — Source data Fig. 2 [file 44318_2026_716_MOESM3_ESM.zip › SD Figure 2/SNX-BAR_25nM-Retromer-mClover_merged.tif]

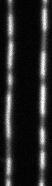

Supplement: Supplementary file 4 — Source data Fig. 3 [file 44318_2026_716_MOESM4_ESM.zip › SD Figure 3/SNX-BAR-GFP_Retromer_Cy5-5-PE.tif]

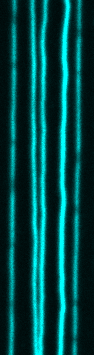

Supplement: Supplementary file 4 — Source data Fig. 3 [file 44318_2026_716_MOESM4_ESM.zip › SD Figure 3/Cy5-5_PE.tif]

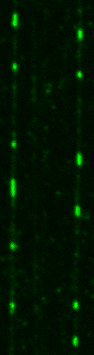

Supplement: Supplementary file 4 — Source data Fig. 3 [file 44318_2026_716_MOESM4_ESM.zip › SD Figure 3/SNX-BAR-GFP.tif]

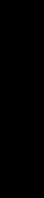

Supplement: Supplementary file 4 — Source data Fig. 3 [file 44318_2026_716_MOESM4_ESM.zip › SD Figure 3/mScarletI-SNX3_Retromer_GFP.tif]

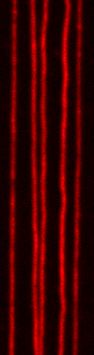

Supplement: Supplementary file 4 — Source data Fig. 3 [file 44318_2026_716_MOESM4_ESM.zip › SD Figure 3/mScarletI-SNX3.tif]

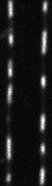

Supplement: Supplementary file 4 — Source data Fig. 3 [file 44318_2026_716_MOESM4_ESM.zip › SD Figure 3/SNX-BAR-GFP_Retromer_GFP.tif]

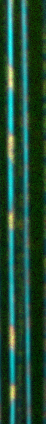

Supplement: Supplementary file 4 — Source data Fig. 3 [file 44318_2026_716_MOESM4_ESM.zip › SD Figure 3/Merged.tif]

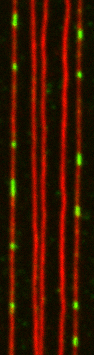

Supplement: Supplementary file 4 — Source data Fig. 3 [file 44318_2026_716_MOESM4_ESM.zip › SD Figure 3/Merged_mScarletI_SNX-BAR-GFP.tif]

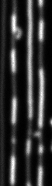

Supplement: Supplementary file 4 — Source data Fig. 3 [file 44318_2026_716_MOESM4_ESM.zip › SD Figure 3/mScarletI-SNX3_SNX-BAR-GFP_Retromer_Cy5-5_PE.tif]

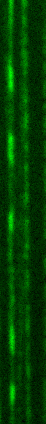

Supplement: Supplementary file 4 — Source data Fig. 3 [file 44318_2026_716_MOESM4_ESM.zip › SD Figure 3/Ear1-488.tif]

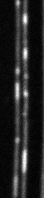

Supplement: Supplementary file 4 — Source data Fig. 3 [file 44318_2026_716_MOESM4_ESM.zip › SD Figure 3/mScarletI-SNX3_Retromer_mSCARLET.tif]

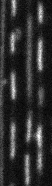

Supplement: Supplementary file 4 — Source data Fig. 3 [file 44318_2026_716_MOESM4_ESM.zip › SD Figure 3/mScarletI-SNX3_SNX-BAR-GFP_Retromer_GFP.tif]

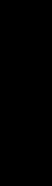

Supplement: Supplementary file 4 — Source data Fig. 3 [file 44318_2026_716_MOESM4_ESM.zip › SD Figure 3/SNX-BAR-GFP_Retromer_mSCARLET.tif]

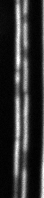

Supplement: Supplementary file 4 — Source data Fig. 3 [file 44318_2026_716_MOESM4_ESM.zip › SD Figure 3/mScarletI-SNX3_Retromer_Cy5-5_PE.tif]

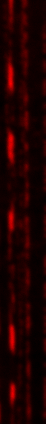

Supplement: Supplementary file 4 — Source data Fig. 3 [file 44318_2026_716_MOESM4_ESM.zip › SD Figure 3/Vps10-546.tif]

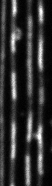

Supplement: Supplementary file 4 — Source data Fig. 3 [file 44318_2026_716_MOESM4_ESM.zip › SD Figure 3/mScarletI-SNX3_SNX-BAR-GFP_Retromer_mSCARLET.tif]

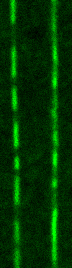

Supplement: Supplementary file 5 — Source data Fig. 4 [file 44318_2026_716_MOESM5_ESM.zip › SD Figure 4/Fig4B_SNX-BAR-GFP.tif]

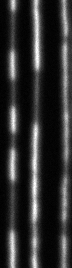

Supplement: Supplementary file 5 — Source data Fig. 4 [file 44318_2026_716_MOESM5_ESM.zip › SD Figure 4/Fig4D_SNX3_coats_add_SNX-BAR-GFP_all_chanels.tif]

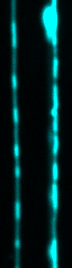

Supplement: Supplementary file 5 — Source data Fig. 4 [file 44318_2026_716_MOESM5_ESM.zip › SD Figure 4/fig4B_Cy5-5_PE.tif]

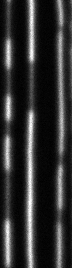

Supplement: Supplementary file 5 — Source data Fig. 4 [file 44318_2026_716_MOESM5_ESM.zip › SD Figure 4/Fig4D_SNX3_coats_all_chanels.tif]

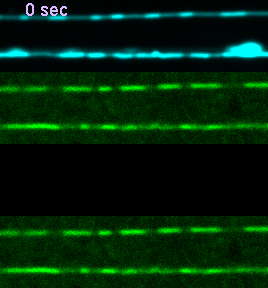

Supplement: Supplementary file 5 — Source data Fig. 4 [file 44318_2026_716_MOESM5_ESM.zip › SD Figure 4/Fig4B_All_channel_and_merged_labelled.tif]

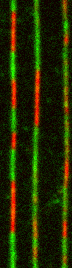

Supplement: Supplementary file 5 — Source data Fig. 4 [file 44318_2026_716_MOESM5_ESM.zip › SD Figure 4/Fig4D_SNX3_coats_add_SNX-BAR-GFP_merged.tif]

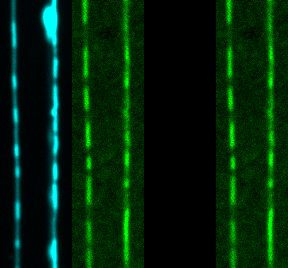

Supplement: Supplementary file 5 — Source data Fig. 4 [file 44318_2026_716_MOESM5_ESM.zip › SD Figure 4/Fig4B_All_channel_and_merged.tif]

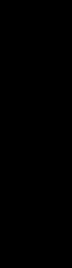

Supplement: Supplementary file 5 — Source data Fig. 4 [file 44318_2026_716_MOESM5_ESM.zip › SD Figure 4/Fig4B_mSCarletI-SNX3.tif]

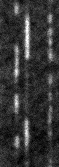

Supplement: Supplementary file 6 — Source data Fig. 5 [file 44318_2026_716_MOESM6_ESM.zip › SD Figure 5/SNX-BAR-GFP.tif]

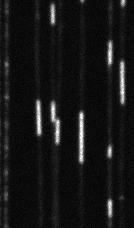

Supplement: Supplementary file 6 — Source data Fig. 5 [file 44318_2026_716_MOESM6_ESM.zip › SD Figure 5/1-2_SNX-BAR-SNX3.tif]

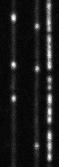

Supplement: Supplementary file 6 — Source data Fig. 5 [file 44318_2026_716_MOESM6_ESM.zip › SD Figure 5/GFP-SNX3.tif]

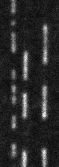

Supplement: Supplementary file 6 — Source data Fig. 5 [file 44318_2026_716_MOESM6_ESM.zip › SD Figure 5/SNX-BAR-GFP_SNX3.tif]

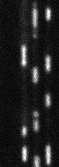

Supplement: Supplementary file 6 — Source data Fig. 5 [file 44318_2026_716_MOESM6_ESM.zip › SD Figure 5/GFP-SNX3_SNX-BAR.tif]

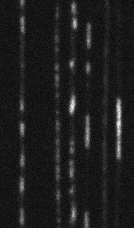

Supplement: Supplementary file 6 — Source data Fig. 5 [file 44318_2026_716_MOESM6_ESM.zip › SD Figure 5/2-1_SNX-BAR-SNX3.tif]

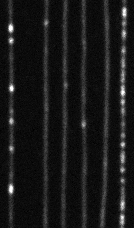

Supplement: Supplementary file 6 — Source data Fig. 5 [file 44318_2026_716_MOESM6_ESM.zip › SD Figure 5/SNX3.tif]

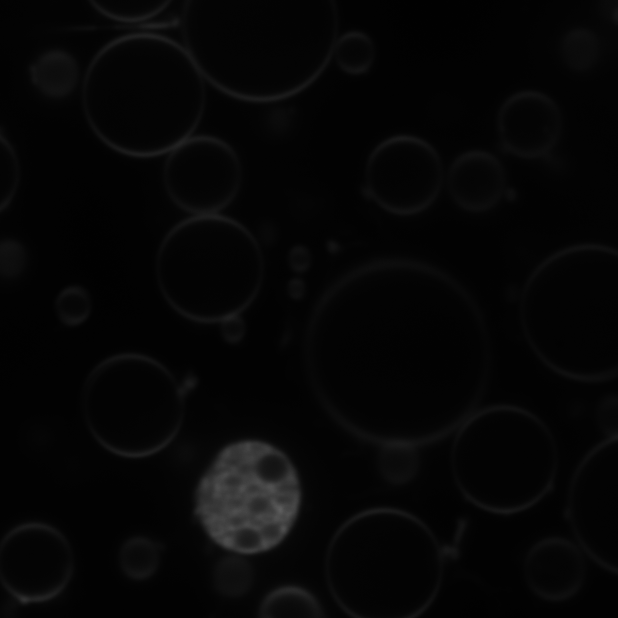

Supplement: Supplementary file 7 — Source data Fig. 6 [file 44318_2026_716_MOESM7_ESM.zip › SD Figure 6/SNX-BAR-GFP_Retromer.tif]

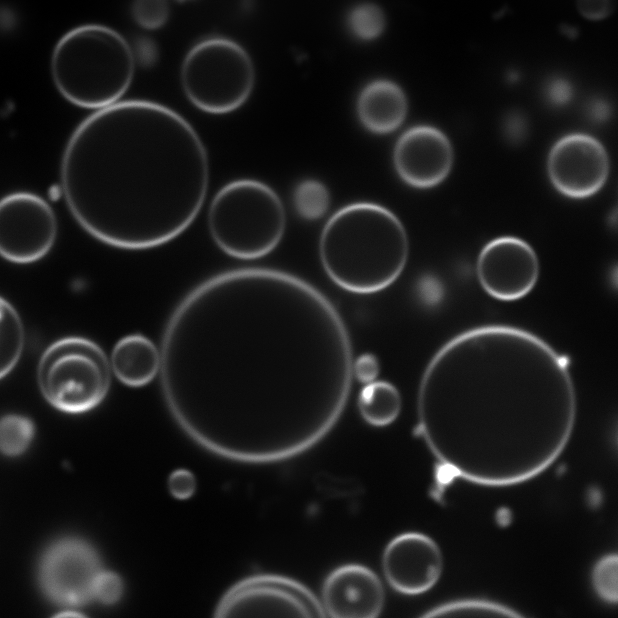

Supplement: Supplementary file 7 — Source data Fig. 6 [file 44318_2026_716_MOESM7_ESM.zip › SD Figure 6/GUVs_only.tif]

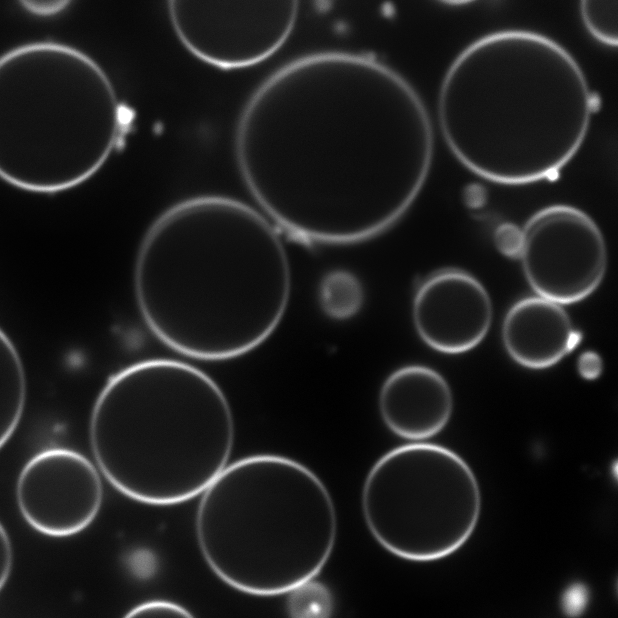

Supplement: Supplementary file 7 — Source data Fig. 6 [file 44318_2026_716_MOESM7_ESM.zip › SD Figure 6/mScraletI-SNX3_Retromer.tif]

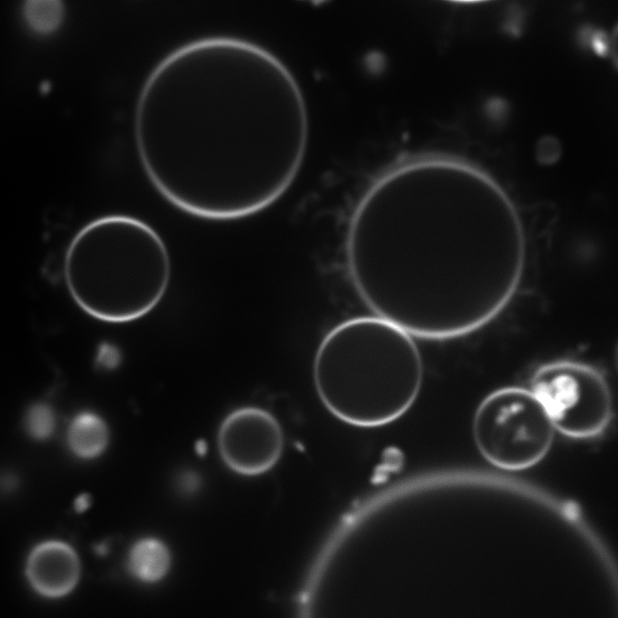

Supplement: Supplementary file 7 — Source data Fig. 6 [file 44318_2026_716_MOESM7_ESM.zip › SD Figure 6/mScarletI-SNX3_SNX-BAR-GFP_Retromer.tif]

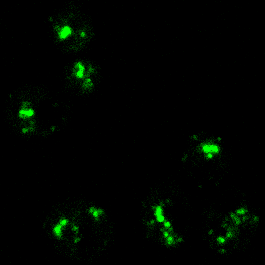

Supplement: Supplementary file 8 — Source data Fig. 7 [file 44318_2026_716_MOESM8_ESM.zip › SD Figure 7/C4-MAX_Vps5_17007.1.tif]

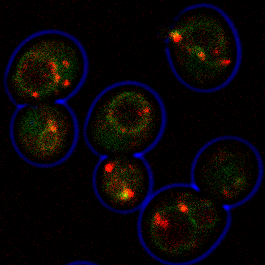

Supplement: Supplementary file 8 — Source data Fig. 7 [file 44318_2026_716_MOESM8_ESM.zip › SD Figure 7/C2-MAX_Vps5_Snx3_vps35KO011.1.tif]

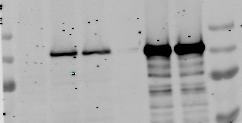

Supplement: Supplementary file 8 — Source data Fig. 7 [file 44318_2026_716_MOESM8_ESM.zip › SD Figure 7/Vps35mCherry IP.jpg]

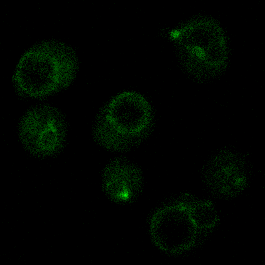

Supplement: Supplementary file 8 — Source data Fig. 7 [file 44318_2026_716_MOESM8_ESM.zip › SD Figure 7/C4-MAX_Vps5_Snx3_vps35KO011.1.tif]

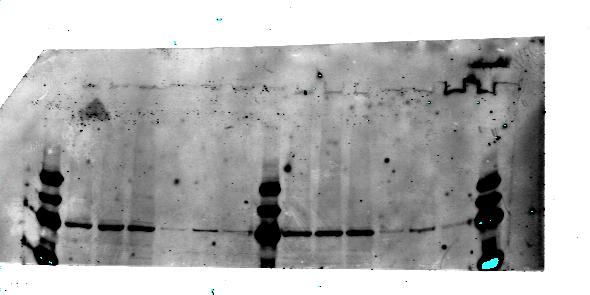

Supplement: Supplementary file 8 — Source data Fig. 7 [file 44318_2026_716_MOESM8_ESM.zip › SD Figure 7/Vps5-HA.jpg]

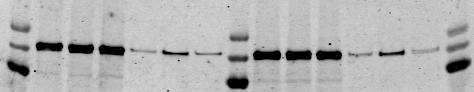

Supplement: Supplementary file 8 — Source data Fig. 7 [file 44318_2026_716_MOESM8_ESM.zip › SD Figure 7/Vps35mCherry.jpg]

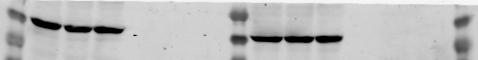

Supplement: Supplementary file 8 — Source data Fig. 7 [file 44318_2026_716_MOESM8_ESM.zip › SD Figure 7/Pgk1_1.jpg]

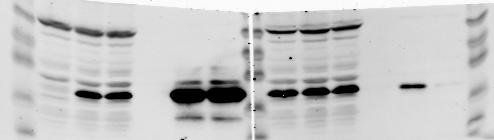

Supplement: Supplementary file 8 — Source data Fig. 7 [file 44318_2026_716_MOESM8_ESM.zip › SD Figure 7/Pgk1 for Vps35 IP.jpg]

Vps35-mCherry

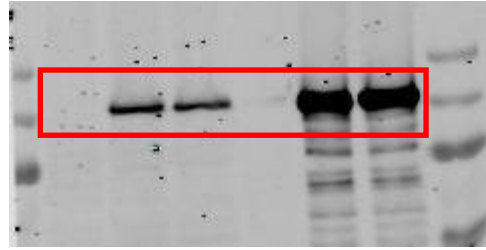

Snx3-V5

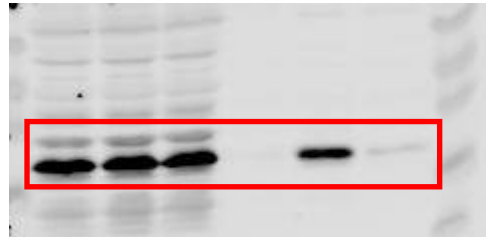

Vps5-HA

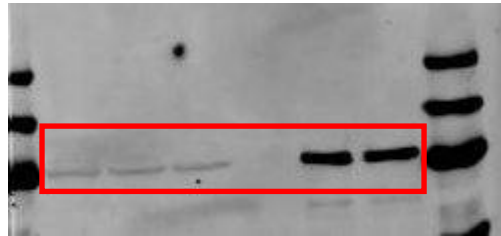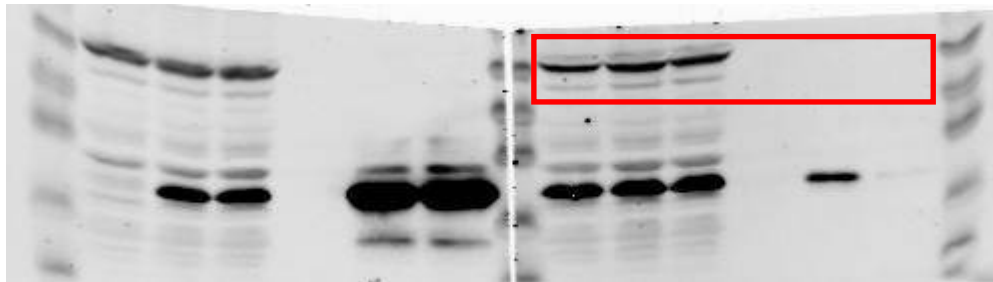

Pgk1

Supplement: Supplementary file 8 — Source data Fig. 7 [file 44318_2026_716_MOESM8_ESM.zip › SD Figure 7/Fig7C_blots.pdf]

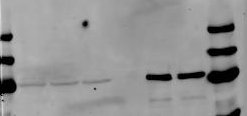

Supplement: Supplementary file 8 — Source data Fig. 7 [file 44318_2026_716_MOESM8_ESM.zip › SD Figure 7/Vps5 for Vps35 IP.jpg]

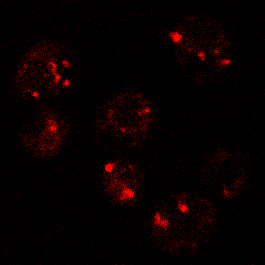

Supplement: Supplementary file 8 — Source data Fig. 7 [file 44318_2026_716_MOESM8_ESM.zip › SD Figure 7/C3-MAX_Vps5_Snx3_vps35KO011.1.tif]

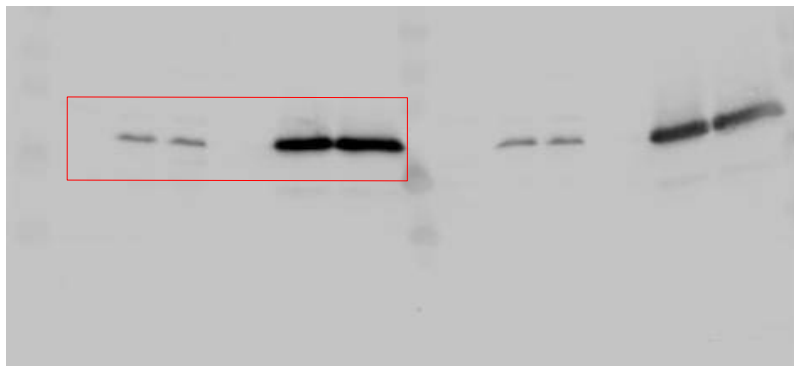

Snx3-V5

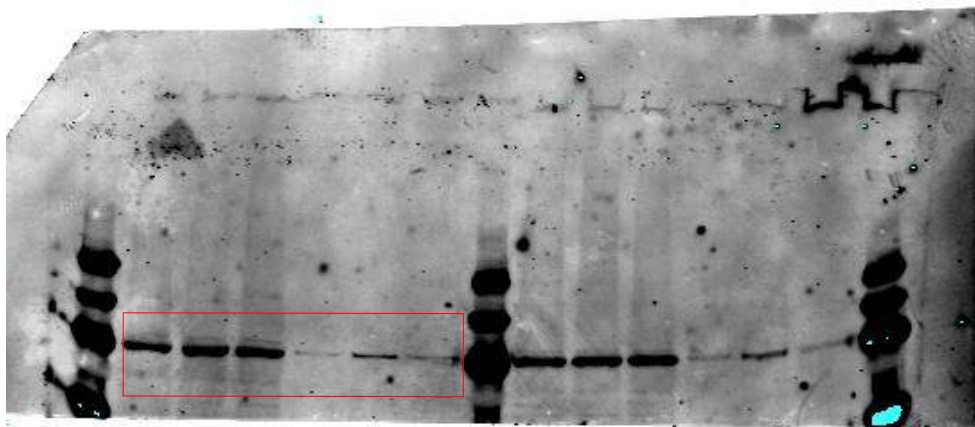

Vps5-HA

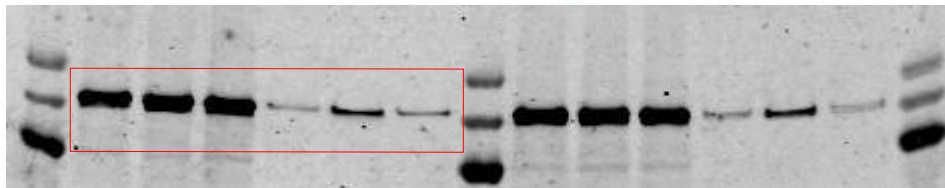

Vps35-mCherry

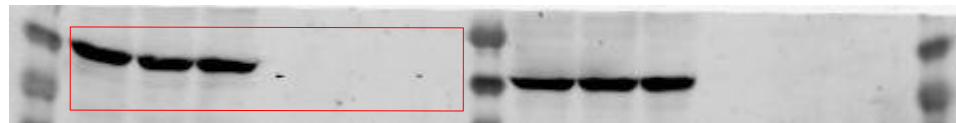

Pgk1

Supplement: Supplementary file 8 — Source data Fig. 7 [file 44318_2026_716_MOESM8_ESM.zip › SD Figure 7/7E_Blots.pdf]

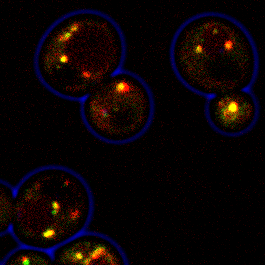

Supplement: Supplementary file 8 — Source data Fig. 7 [file 44318_2026_716_MOESM8_ESM.zip › SD Figure 7/C2-MAX_Vps5_17_35KO012.1.tif]

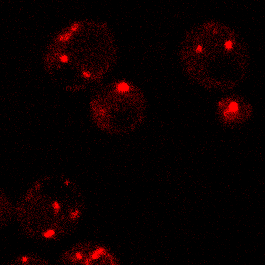

Supplement: Supplementary file 8 — Source data Fig. 7 [file 44318_2026_716_MOESM8_ESM.zip › SD Figure 7/C3-MAX_Vps5_17_35KO012.1.tif]

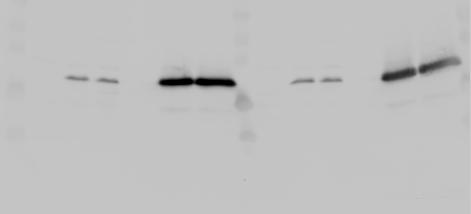

Supplement: Supplementary file 8 — Source data Fig. 7 [file 44318_2026_716_MOESM8_ESM.zip › SD Figure 7/Snx3-V5.jpg]

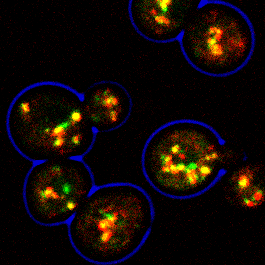

Supplement: Supplementary file 8 — Source data Fig. 7 [file 44318_2026_716_MOESM8_ESM.zip › SD Figure 7/C2-MAX_Vps5_Snx3008.1.tif]

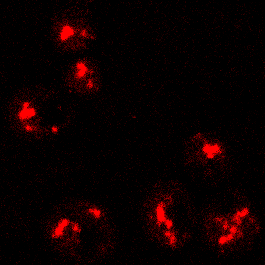

Supplement: Supplementary file 8 — Source data Fig. 7 [file 44318_2026_716_MOESM8_ESM.zip › SD Figure 7/C3-MAX_Vps5_17007.1.tif]

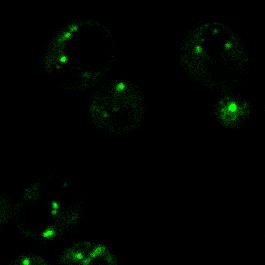

Supplement: Supplementary file 8 — Source data Fig. 7 [file 44318_2026_716_MOESM8_ESM.zip › SD Figure 7/C4-MAX_Vps5_17_35KO012.1.tif]

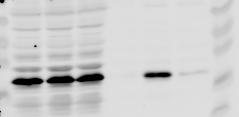

Supplement: Supplementary file 8 — Source data Fig. 7 [file 44318_2026_716_MOESM8_ESM.zip › SD Figure 7/Snx3 for Vps35 IP.jpg]

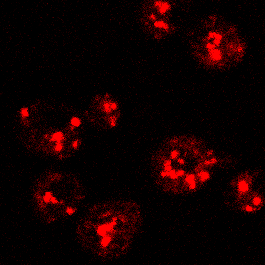

Supplement: Supplementary file 8 — Source data Fig. 7 [file 44318_2026_716_MOESM8_ESM.zip › SD Figure 7/C3-MAX_Vps5_Snx3008.1.tif]

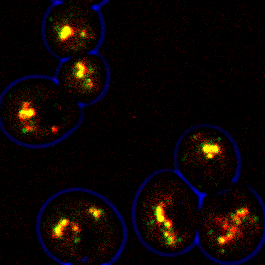

Supplement: Supplementary file 8 — Source data Fig. 7 [file 44318_2026_716_MOESM8_ESM.zip › SD Figure 7/C2-MAX_Vps5_17007.1.tif]

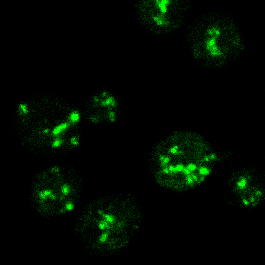

Supplement: Supplementary file 8 — Source data Fig. 7 [file 44318_2026_716_MOESM8_ESM.zip › SD Figure 7/C4-MAX_Vps5_Snx3008.1.tif]

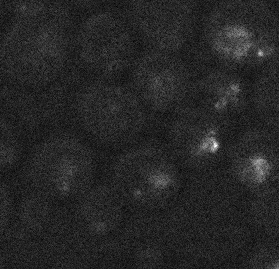

Supplement: Supplementary file 9 — Source data Fig. 8 [file 44318_2026_716_MOESM9_ESM.zip › SD Figure 8/Fig8E_snx3_KO.tif]

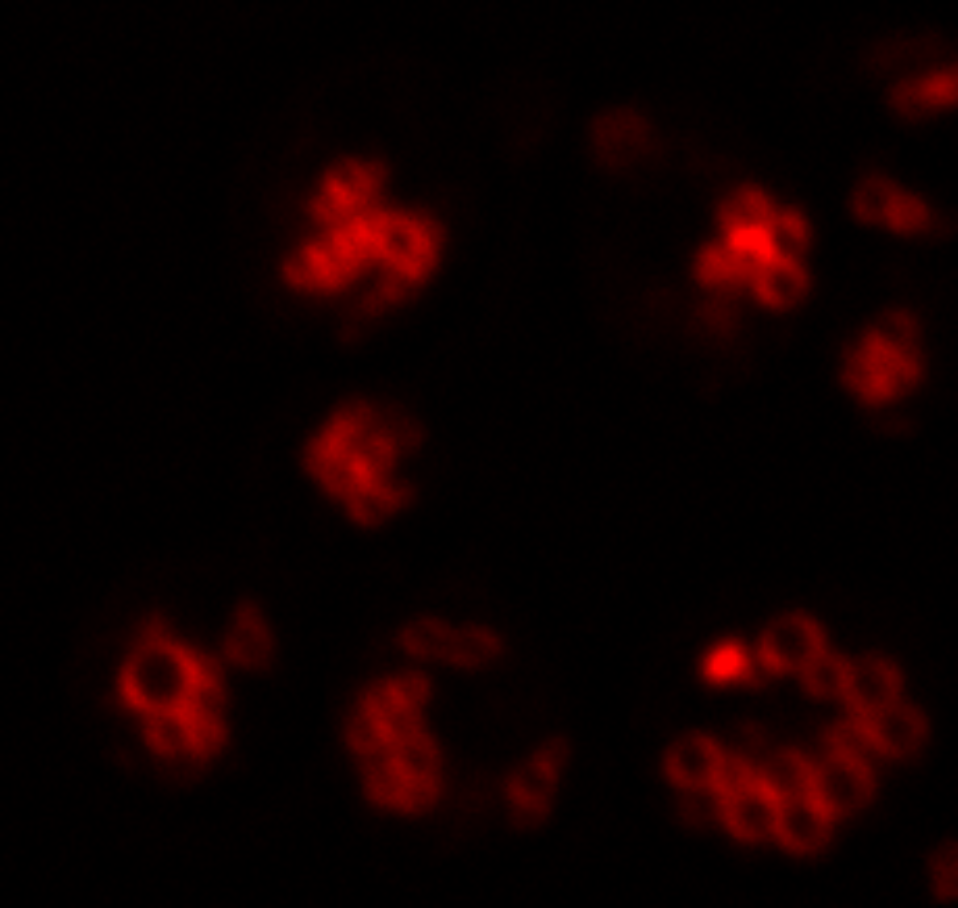

Supplement: Supplementary file 9 — Source data Fig. 8 [file 44318_2026_716_MOESM9_ESM.zip › SD Figure 8/AVG_C2-Vps5-KO_3-2.tif]

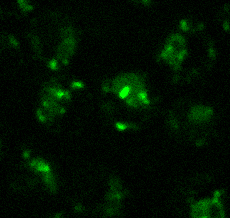

Supplement: Supplementary file 9 — Source data Fig. 8 [file 44318_2026_716_MOESM9_ESM.zip › SD Figure 8/AVG_C1-Ear1-1.tif]

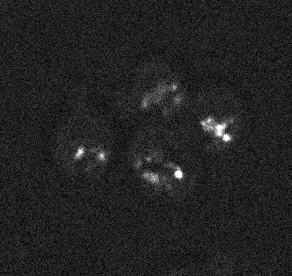

Supplement: Supplementary file 9 — Source data Fig. 8 [file 44318_2026_716_MOESM9_ESM.zip › SD Figure 8/Fig8A_ctrl.tif]

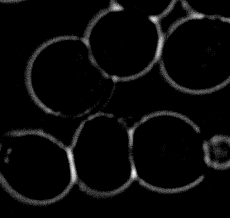

Supplement: Supplementary file 9 — Source data Fig. 8 [file 44318_2026_716_MOESM9_ESM.zip › SD Figure 8/AVG_C3-Ear_Vps5-KO001.tif]
