# Supplementary figures and images for: Hybrid endosomal coats contain different classes of sorting nexins (part 2 of 2)
Source: EMBO J. 2026 Feb 16;45(7):2278–305. doi: 10.1038/s44318-026-00716-0 (PMC13043683; doi:10.1038/s44318-026-00716-0)

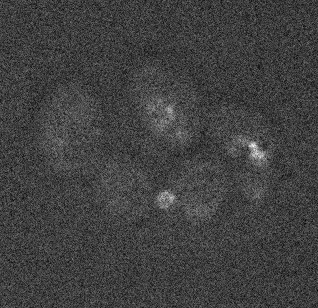

Supplement: Supplementary file 9 — Source data Fig. 8 [file 44318_2026_716_MOESM9_ESM.zip › SD Figure 8/Fig8A_vps17_KO.tif]

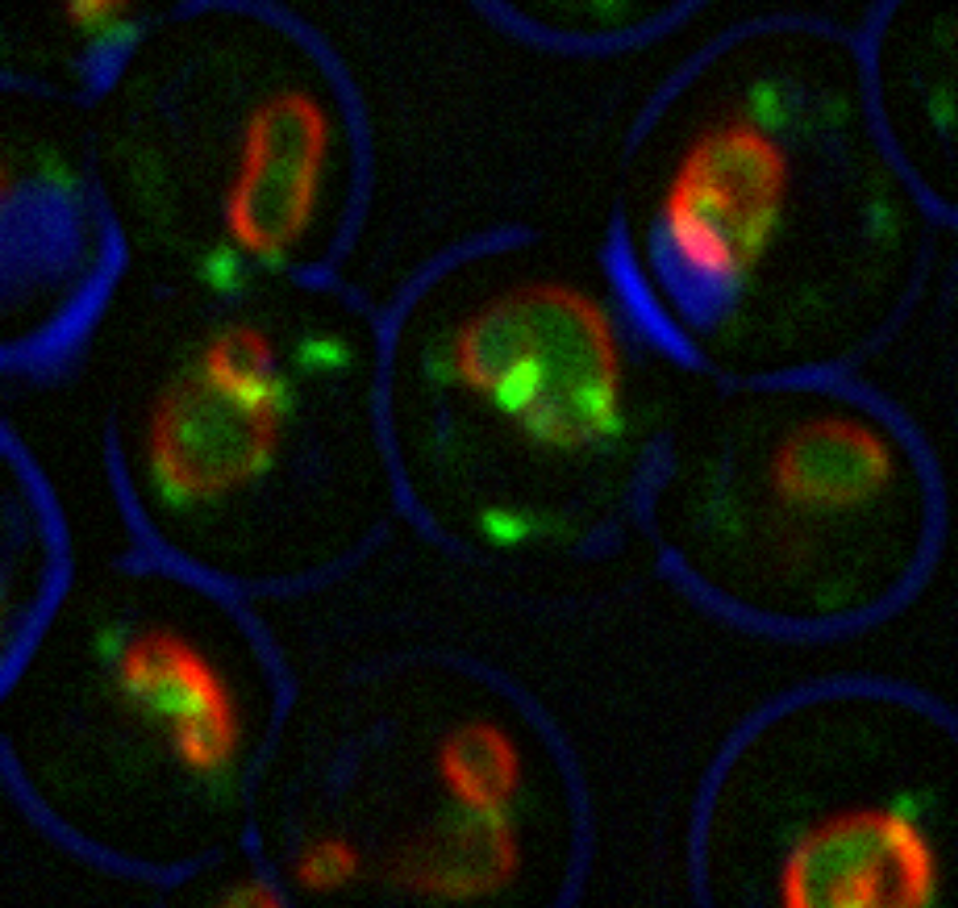

Supplement: Supplementary file 9 — Source data Fig. 8 [file 44318_2026_716_MOESM9_ESM.zip › SD Figure 8/AVG_C4-Ear1-1.tif]

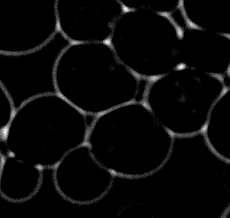

Supplement: Supplementary file 9 — Source data Fig. 8 [file 44318_2026_716_MOESM9_ESM.zip › SD Figure 8/C3-AVG_Ste13_Snx3 KO_1-1.tif]

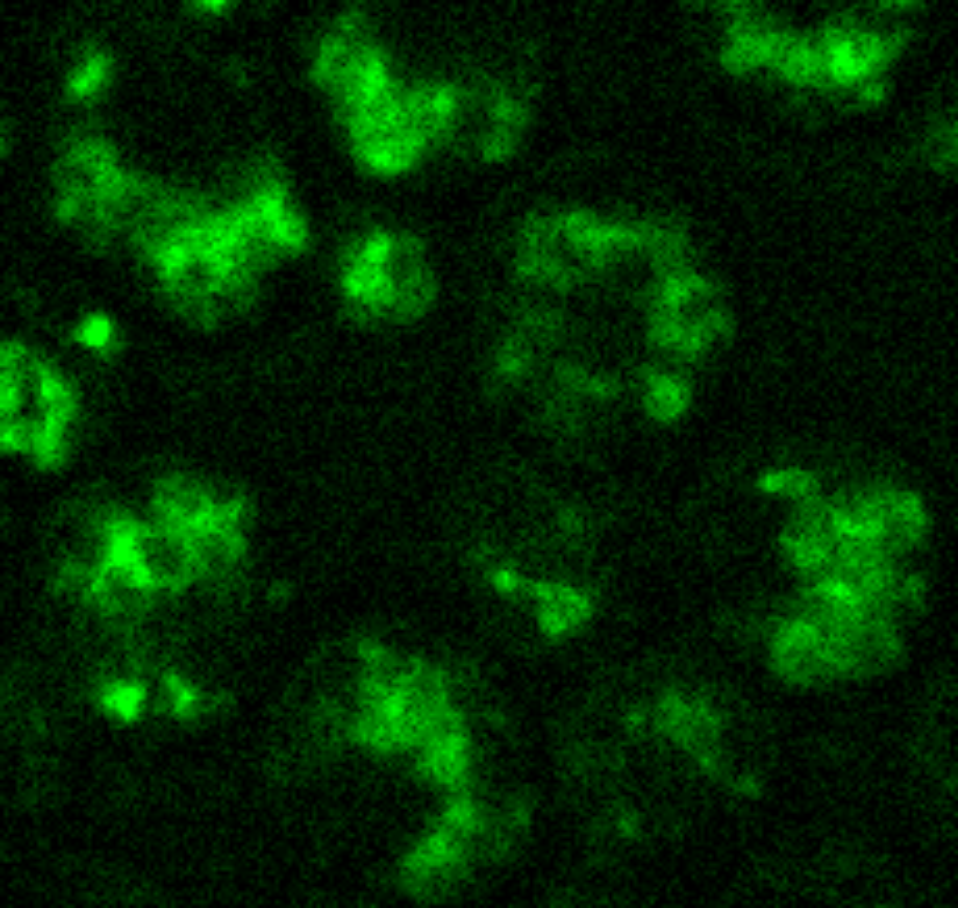

Supplement: Supplementary file 9 — Source data Fig. 8 [file 44318_2026_716_MOESM9_ESM.zip › SD Figure 8/AVG_C1-Ste13_Vps5 KO_1004-1.tif]

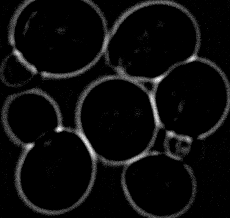

Supplement: Supplementary file 9 — Source data Fig. 8 [file 44318_2026_716_MOESM9_ESM.zip › SD Figure 8/AVG_C3-Ste13_1-1.tif]

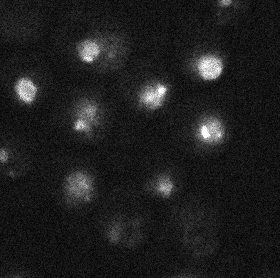

Supplement: Supplementary file 9 — Source data Fig. 8 [file 44318_2026_716_MOESM9_ESM.zip › SD Figure 8/FIg8C_snx3_KO.tif]

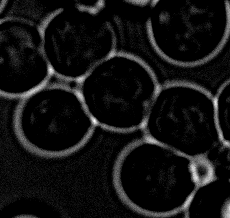

Supplement: Supplementary file 9 — Source data Fig. 8 [file 44318_2026_716_MOESM9_ESM.zip › SD Figure 8/AVG_C3-Ear_Vps17-KO002-1.tif]

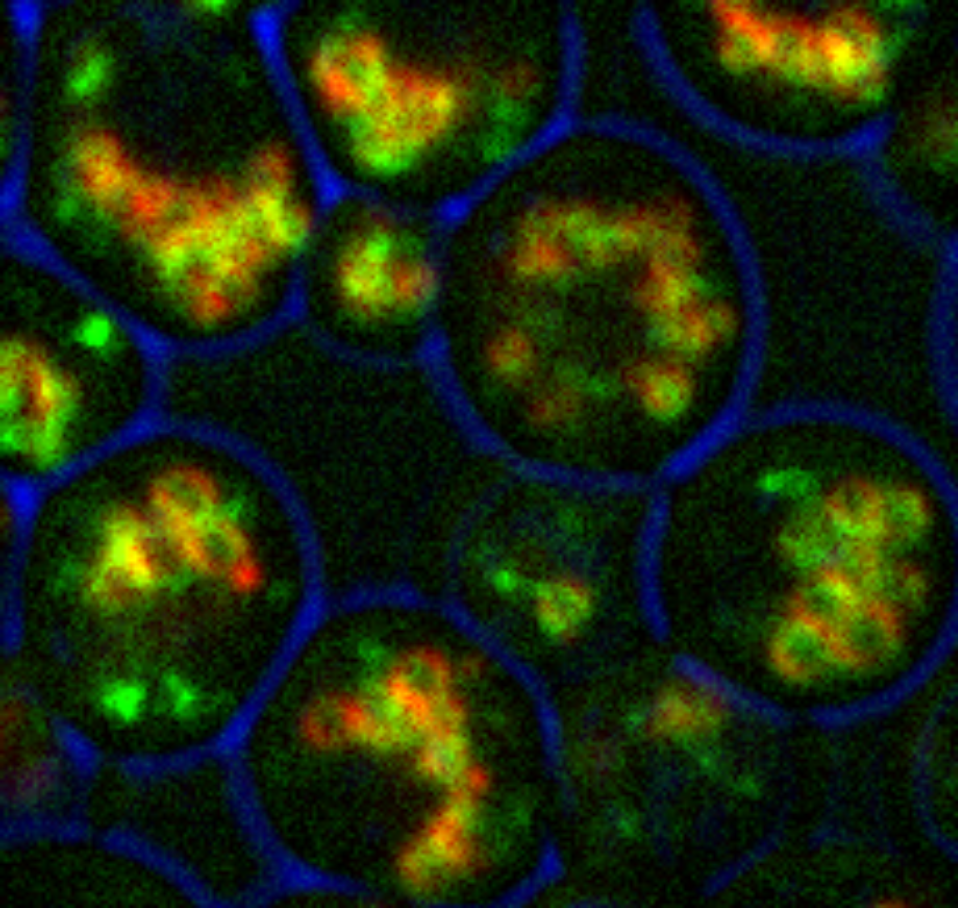

Supplement: Supplementary file 9 — Source data Fig. 8 [file 44318_2026_716_MOESM9_ESM.zip › SD Figure 8/AVG_C4-Ste13_Vps5 KO_1004-1.tif]

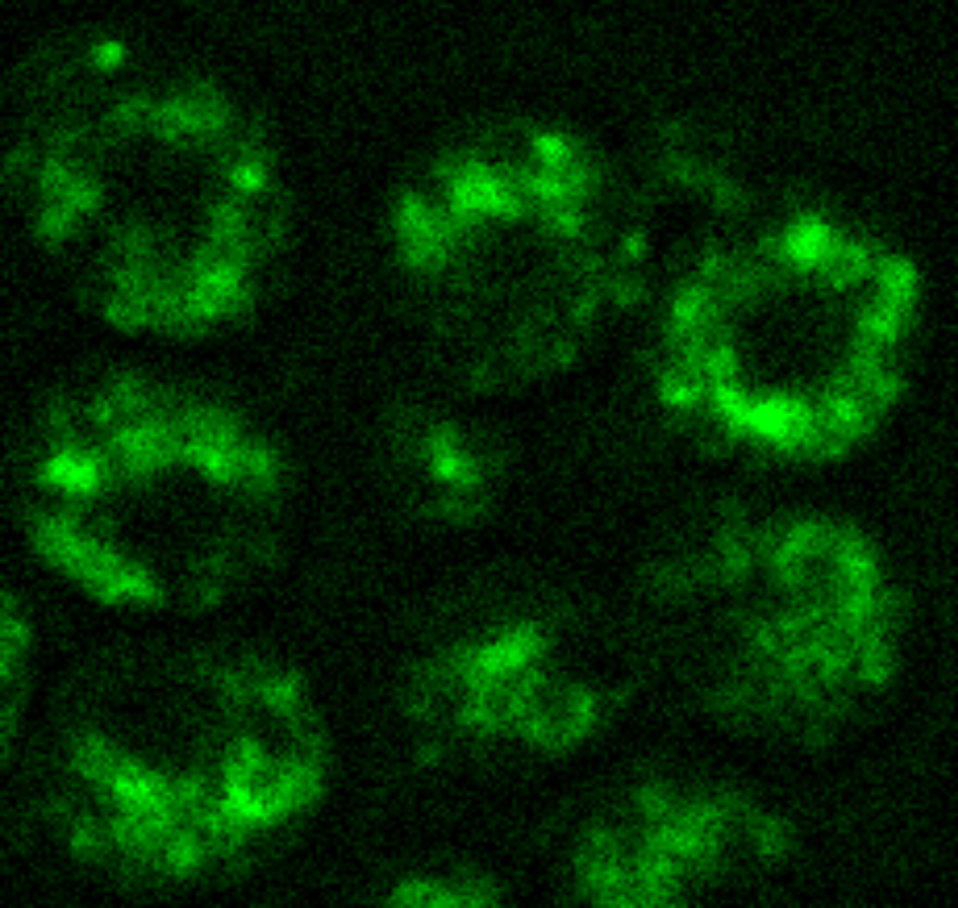

Supplement: Supplementary file 9 — Source data Fig. 8 [file 44318_2026_716_MOESM9_ESM.zip › SD Figure 8/C1-AVG_Ste13_Vps17 KO_1003-1.tif]

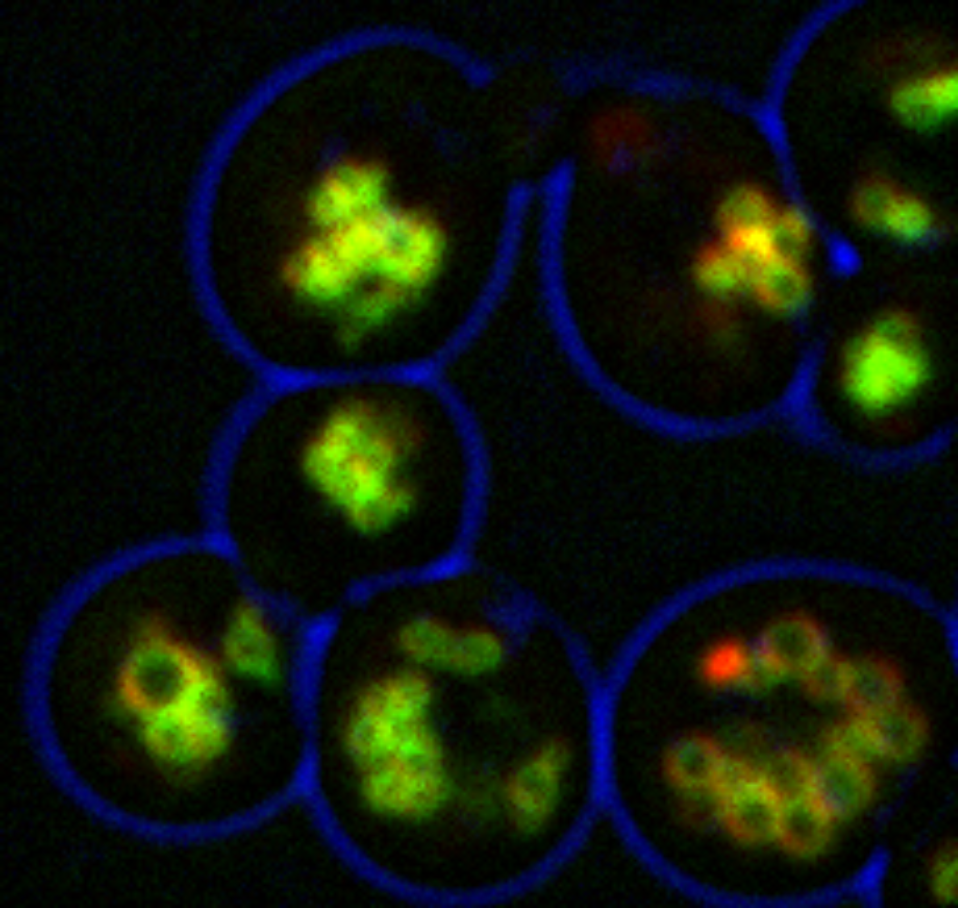

Supplement: Supplementary file 9 — Source data Fig. 8 [file 44318_2026_716_MOESM9_ESM.zip › SD Figure 8/AVG_C4-Vps5-KO_3-2.tif]

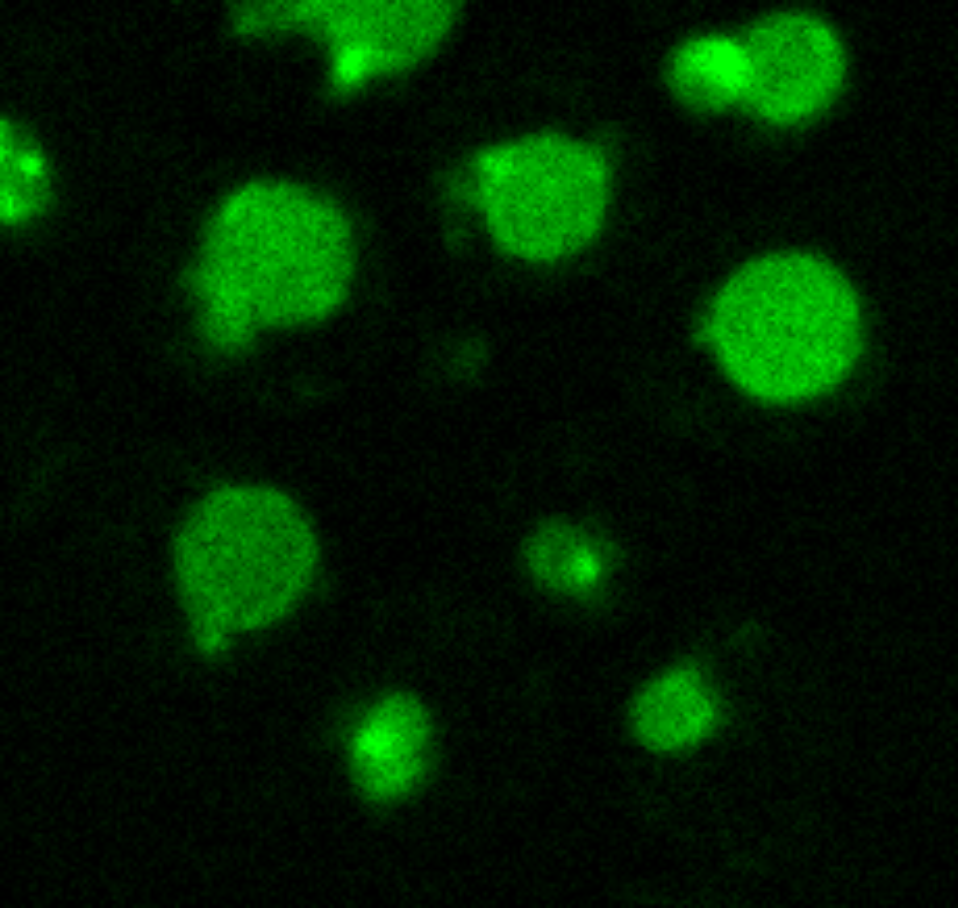

Supplement: Supplementary file 9 — Source data Fig. 8 [file 44318_2026_716_MOESM9_ESM.zip › SD Figure 8/AVG_C1-Ear_Snx3-KO001-1.tif]

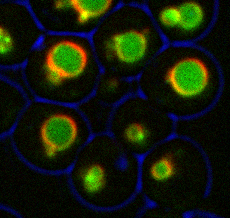

Supplement: Supplementary file 9 — Source data Fig. 8 [file 44318_2026_716_MOESM9_ESM.zip › SD Figure 8/AVG_C4-Ear_Snx3-KO001-1.tif]

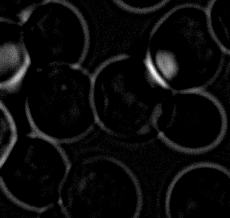

Supplement: Supplementary file 9 — Source data Fig. 8 [file 44318_2026_716_MOESM9_ESM.zip › SD Figure 8/AVG_C3-Ear1-1.tif]

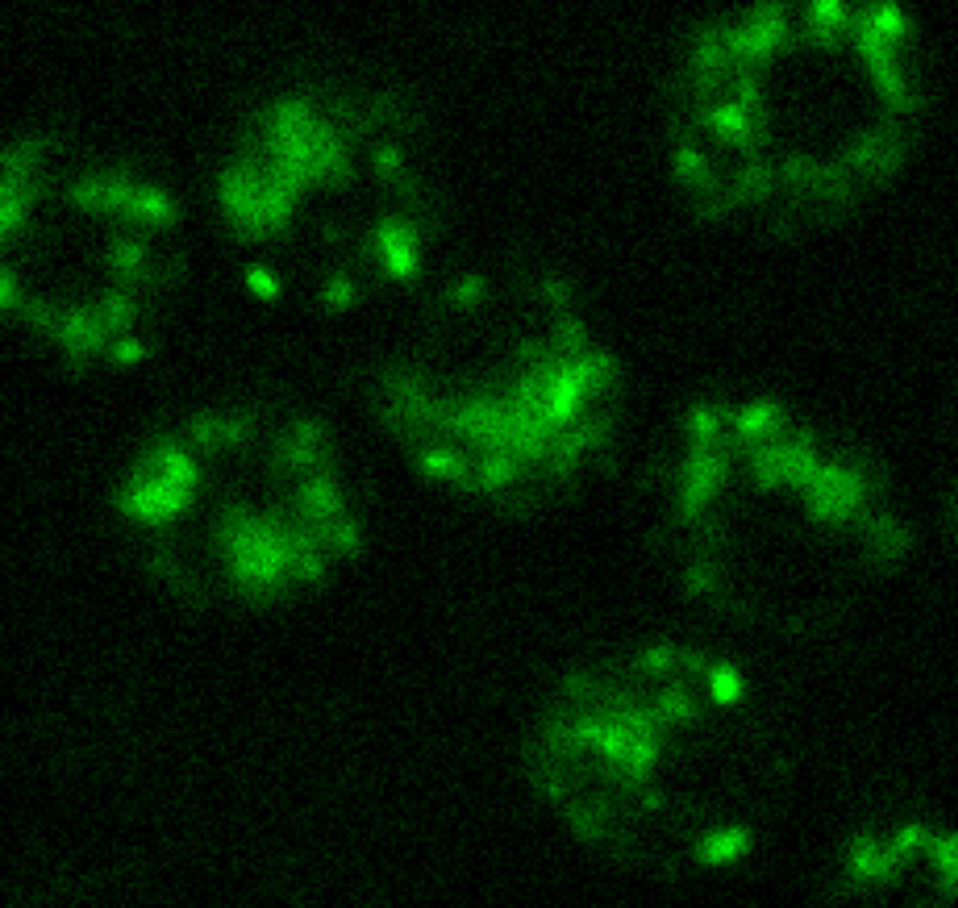

Supplement: Supplementary file 9 — Source data Fig. 8 [file 44318_2026_716_MOESM9_ESM.zip › SD Figure 8/AVG_C1-Ear_Vps17-KO002-1.tif]

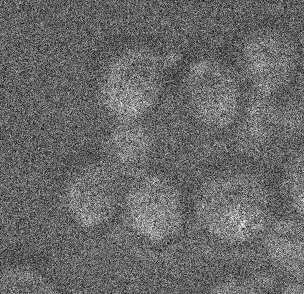

Supplement: Supplementary file 9 — Source data Fig. 8 [file 44318_2026_716_MOESM9_ESM.zip › SD Figure 8/Fig8A_vps5_KO.tif]

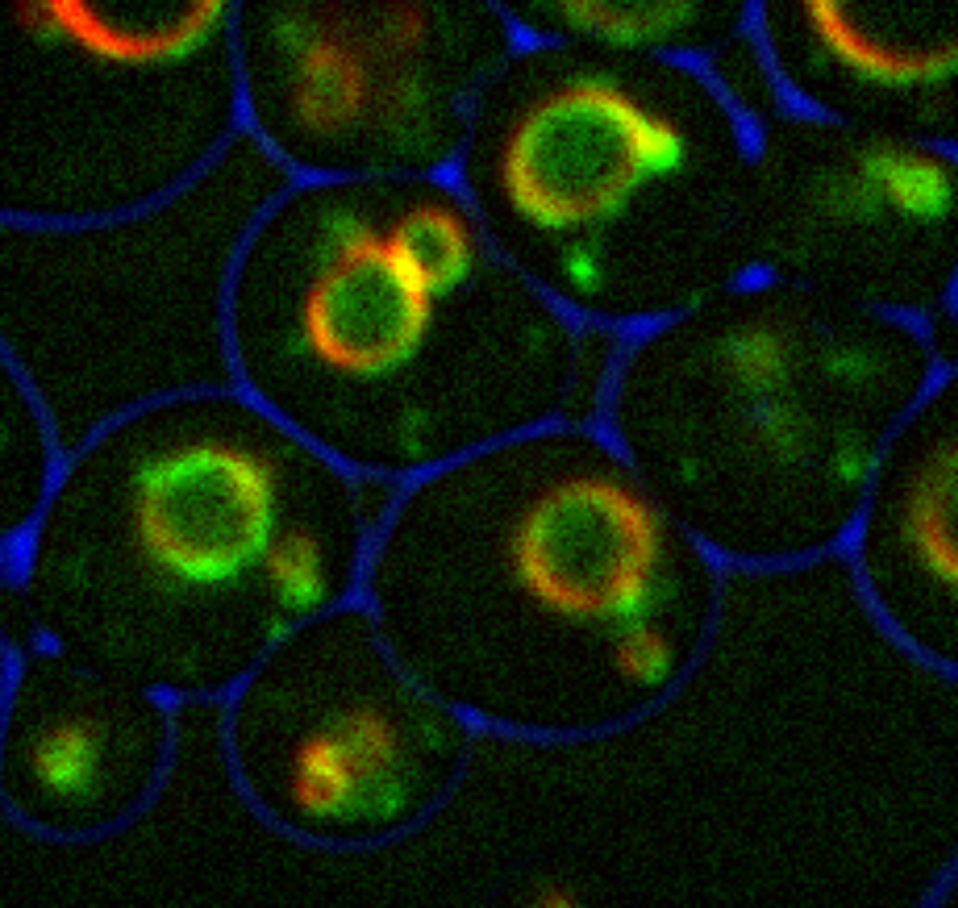

Supplement: Supplementary file 9 — Source data Fig. 8 [file 44318_2026_716_MOESM9_ESM.zip › SD Figure 8/AVG_C4-Ste13_Snx3 KO_1-1.tif]

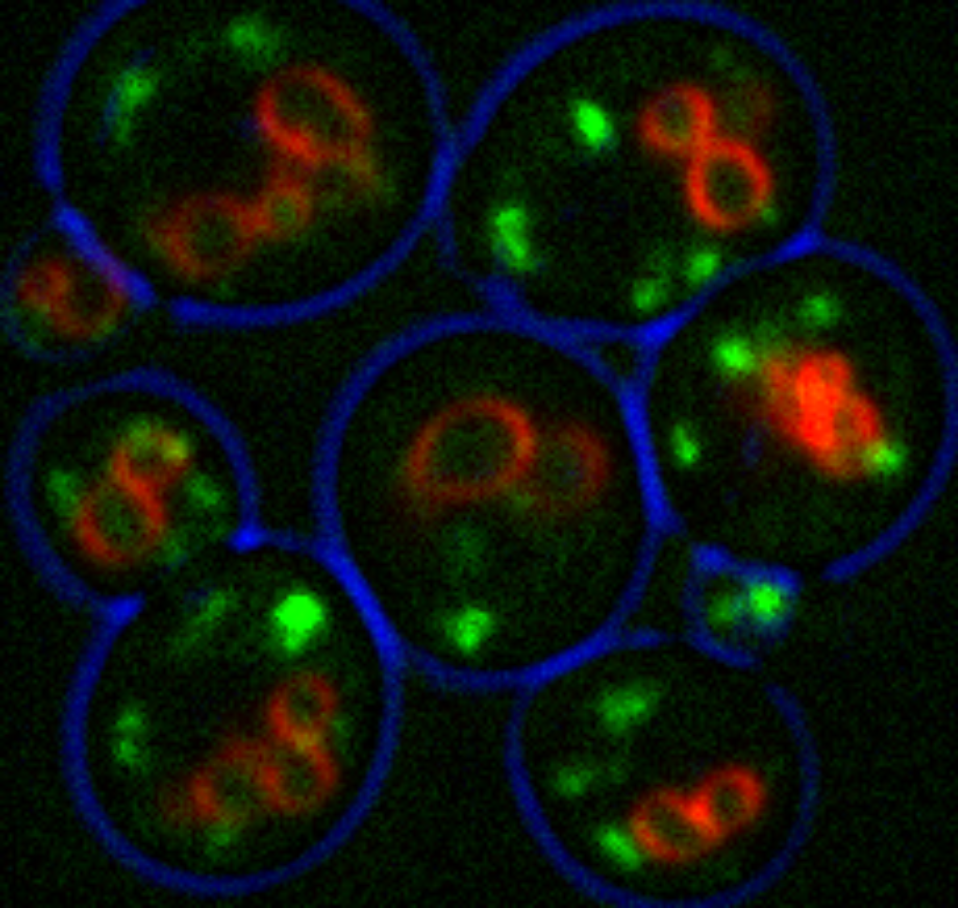

Supplement: Supplementary file 9 — Source data Fig. 8 [file 44318_2026_716_MOESM9_ESM.zip › SD Figure 8/AVG_C4-Ste13_1-1.tif]

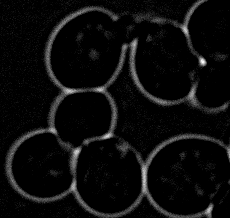

Supplement: Supplementary file 9 — Source data Fig. 8 [file 44318_2026_716_MOESM9_ESM.zip › SD Figure 8/AVG_C3-Vps5-KO_3-1.tif]

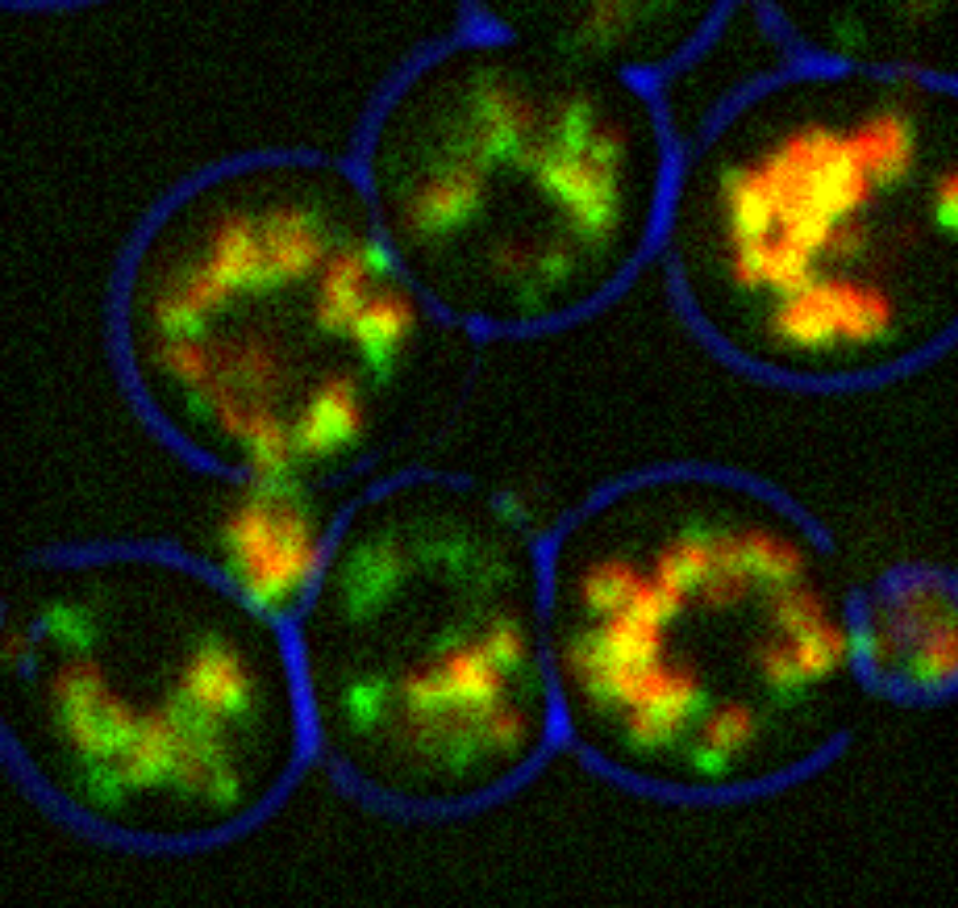

Supplement: Supplementary file 9 — Source data Fig. 8 [file 44318_2026_716_MOESM9_ESM.zip › SD Figure 8/AVG_C4-Ear_Vps5-KO001.tif]

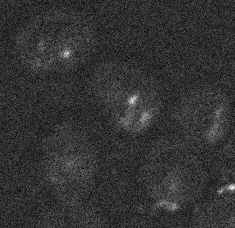

Supplement: Supplementary file 9 — Source data Fig. 8 [file 44318_2026_716_MOESM9_ESM.zip › SD Figure 8/Fig8A_snx3_KO.tif]

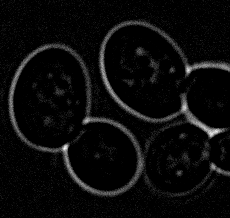

Supplement: Supplementary file 9 — Source data Fig. 8 [file 44318_2026_716_MOESM9_ESM.zip › SD Figure 8/AVG_C3-Vps17-KO_3.tif]

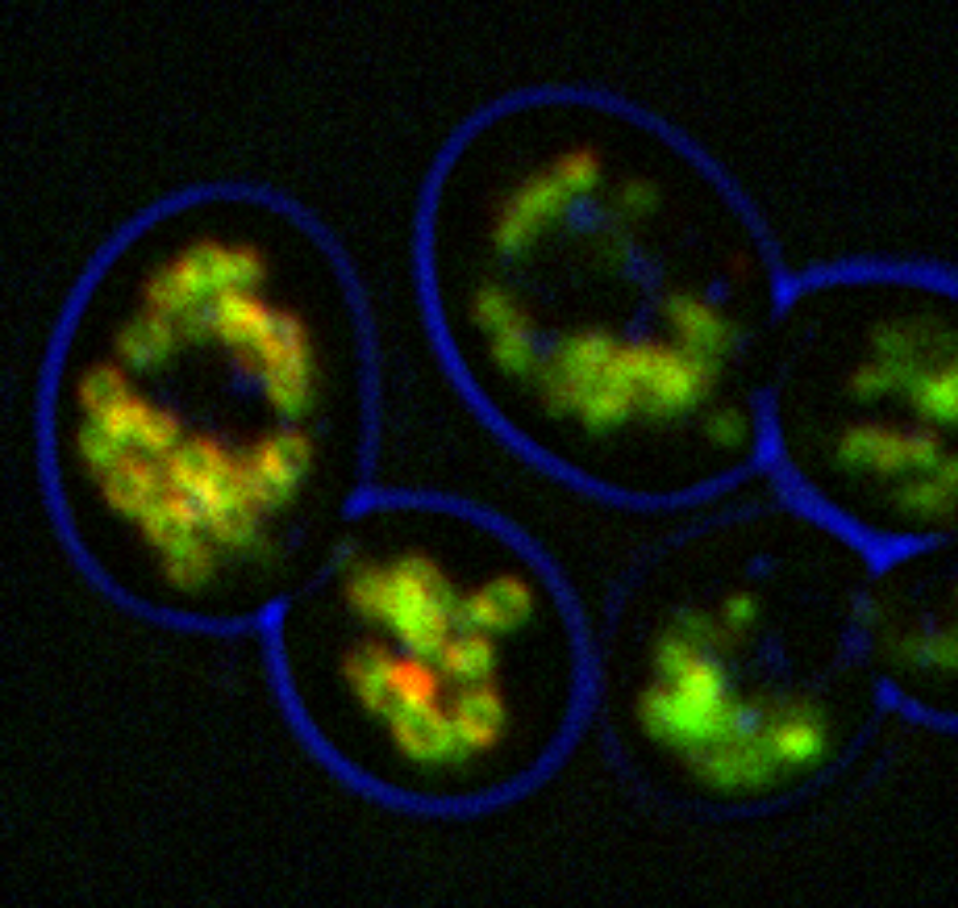

Supplement: Supplementary file 9 — Source data Fig. 8 [file 44318_2026_716_MOESM9_ESM.zip › SD Figure 8/AVG_C4-Vps17-KO_3.tif]

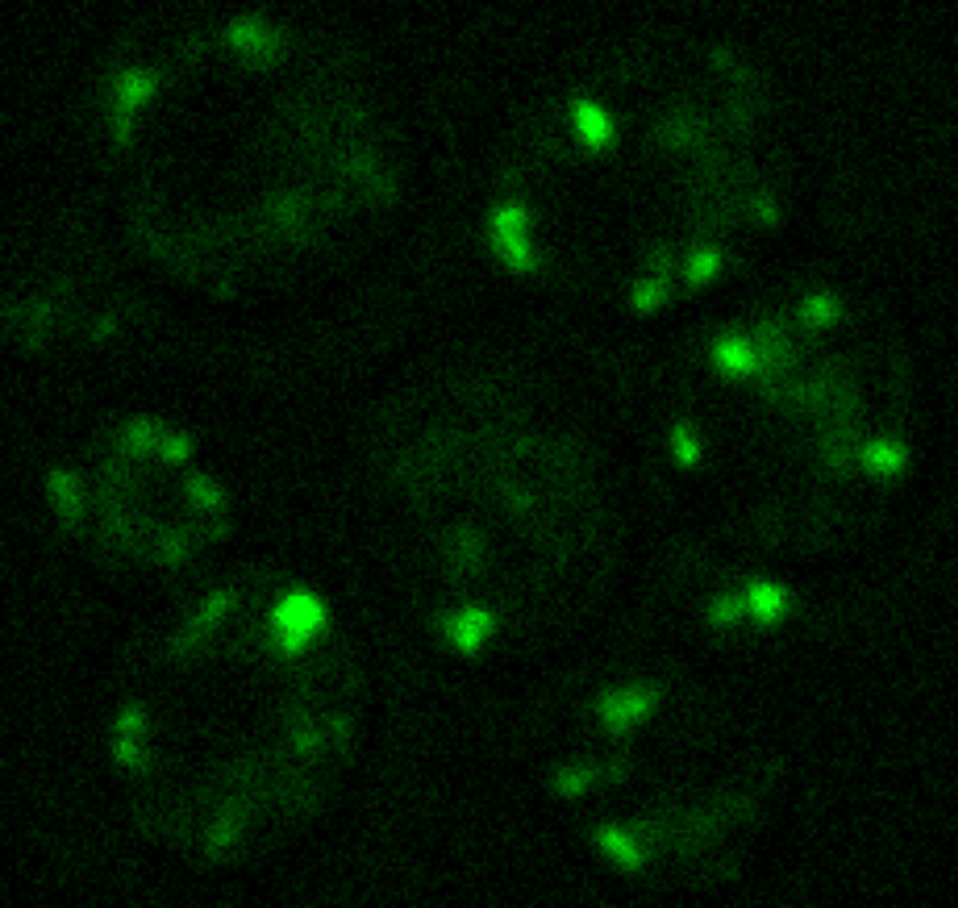

Supplement: Supplementary file 9 — Source data Fig. 8 [file 44318_2026_716_MOESM9_ESM.zip › SD Figure 8/AVG_C1-Ste13_1-1.tif]

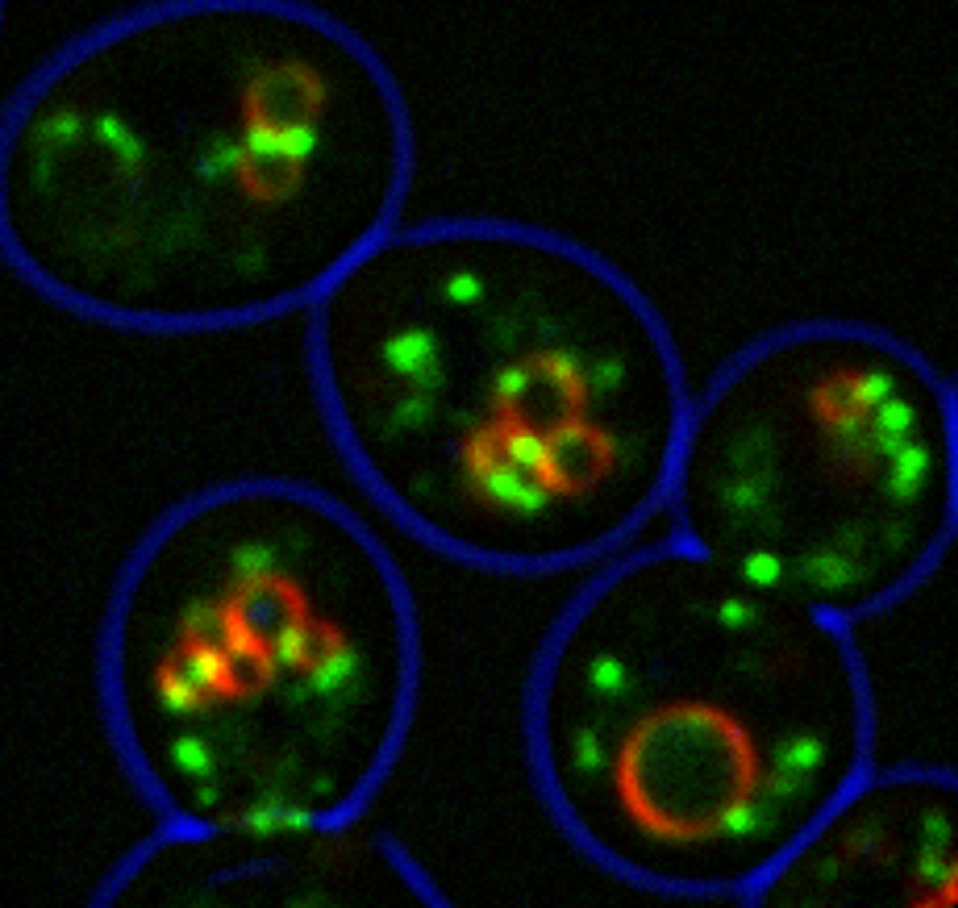

Supplement: Supplementary file 9 — Source data Fig. 8 [file 44318_2026_716_MOESM9_ESM.zip › SD Figure 8/C4-AVG_Sxn3-KO_1-1.tif]

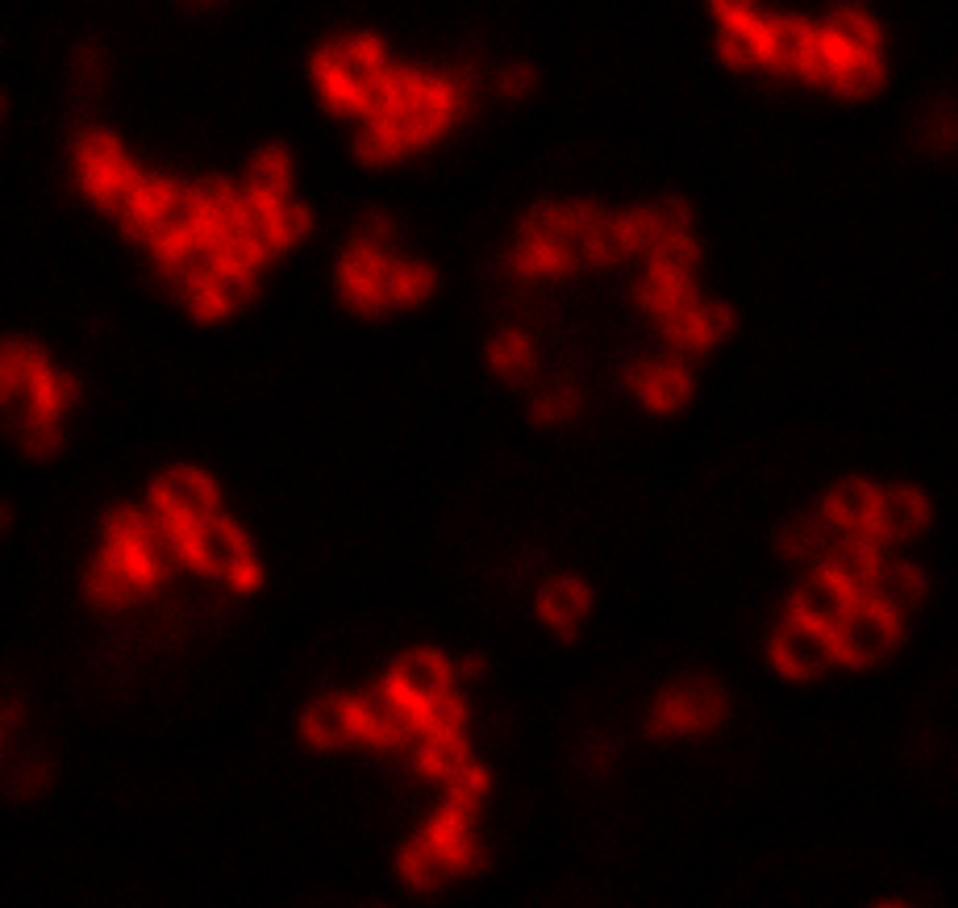

Supplement: Supplementary file 9 — Source data Fig. 8 [file 44318_2026_716_MOESM9_ESM.zip › SD Figure 8/AVG_C2-Ste13_Vps5 KO_1004-1.tif]

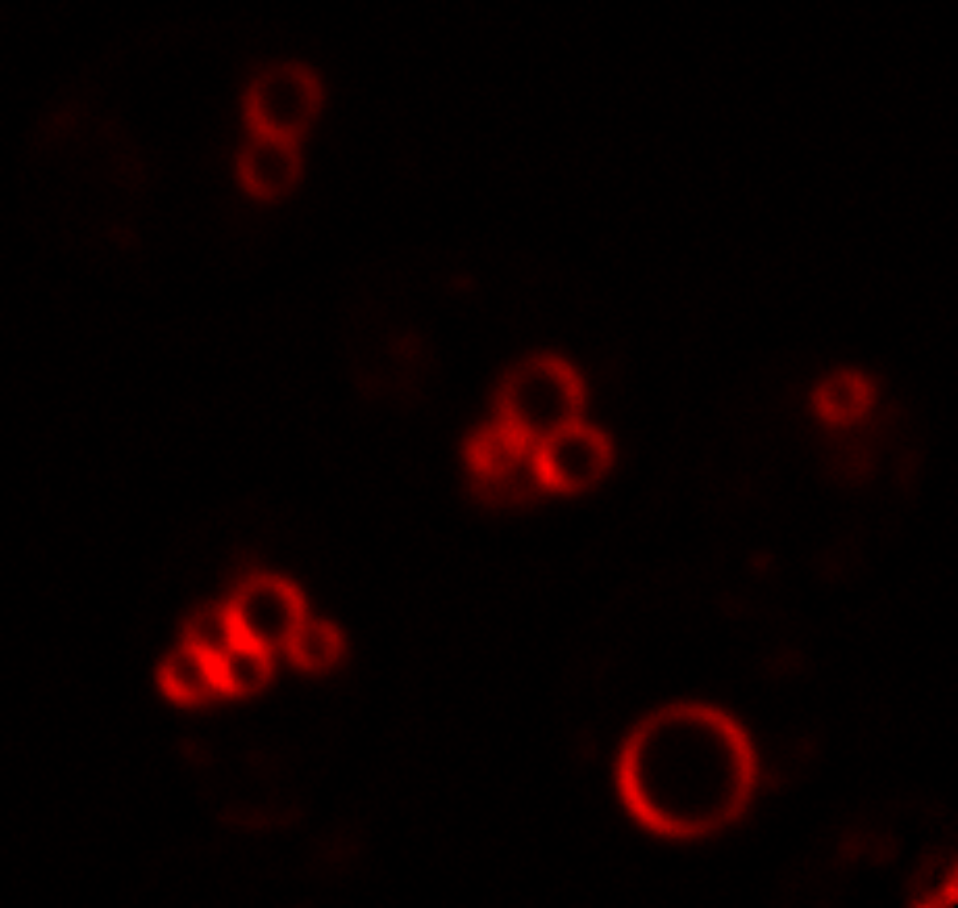

Supplement: Supplementary file 9 — Source data Fig. 8 [file 44318_2026_716_MOESM9_ESM.zip › SD Figure 8/C2-AVG_Sxn3-KO_1-1.tif]

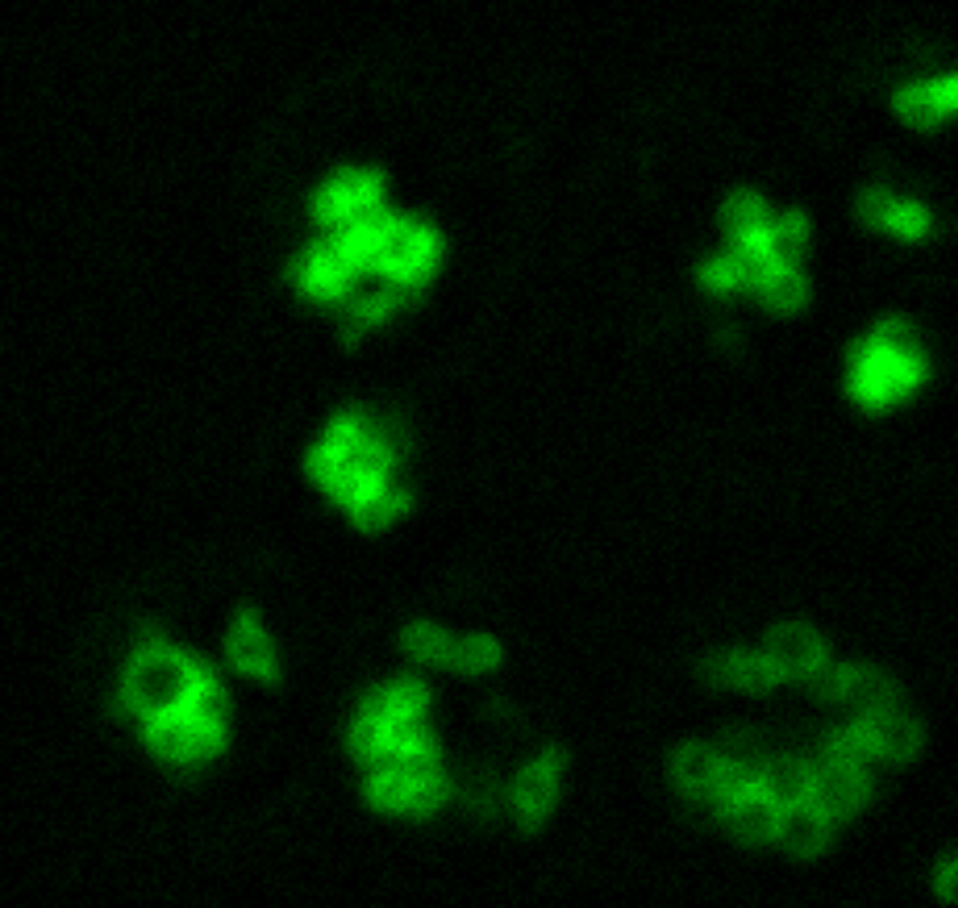

Supplement: Supplementary file 9 — Source data Fig. 8 [file 44318_2026_716_MOESM9_ESM.zip › SD Figure 8/AVG_C1-Vps5-KO_3-2.tif]

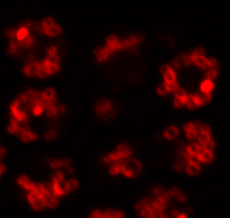

Supplement: Supplementary file 9 — Source data Fig. 8 [file 44318_2026_716_MOESM9_ESM.zip › SD Figure 8/C2-AVG_Ste13_Vps17 KO_1003-1.tif]

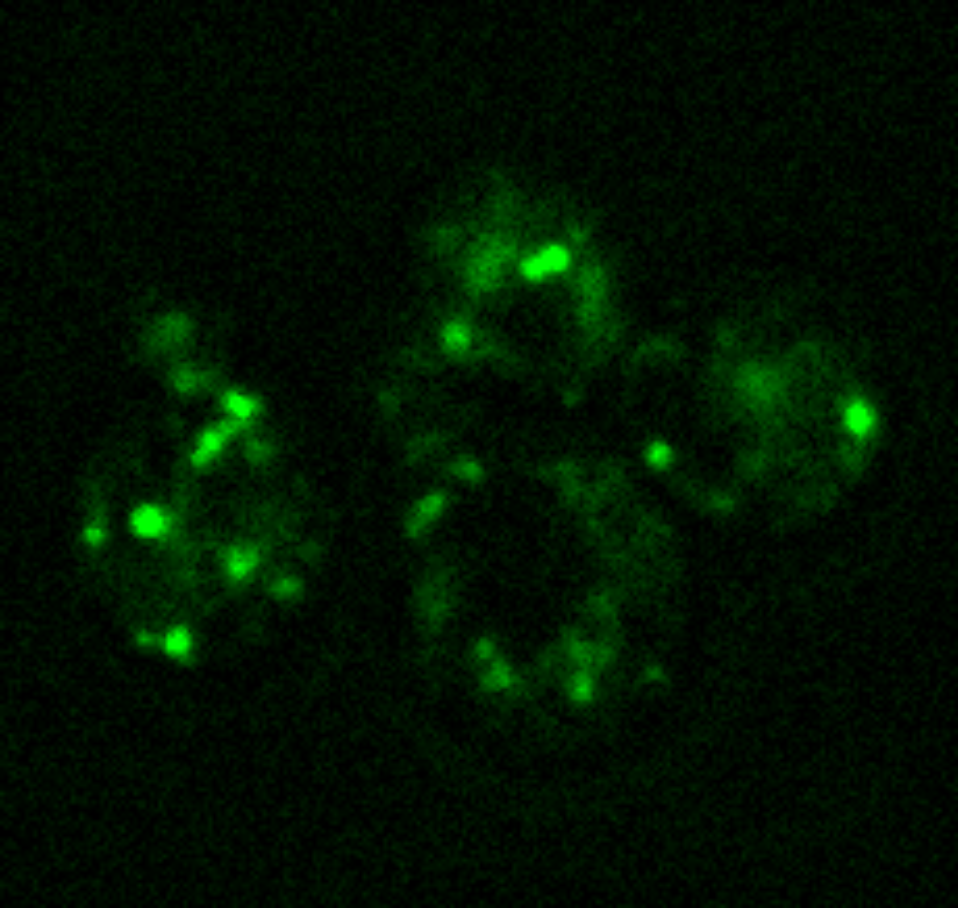

Supplement: Supplementary file 9 — Source data Fig. 8 [file 44318_2026_716_MOESM9_ESM.zip › SD Figure 8/C1-AVG_cont_1.tif]

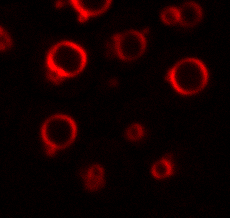

Supplement: Supplementary file 9 — Source data Fig. 8 [file 44318_2026_716_MOESM9_ESM.zip › SD Figure 8/AVG_C2-Ear_Snx3-KO001-1.tif]

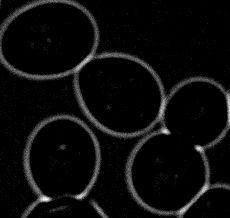

Supplement: Supplementary file 9 — Source data Fig. 8 [file 44318_2026_716_MOESM9_ESM.zip › SD Figure 8/C3-AVG_Sxn3-KO_1-1.tif]

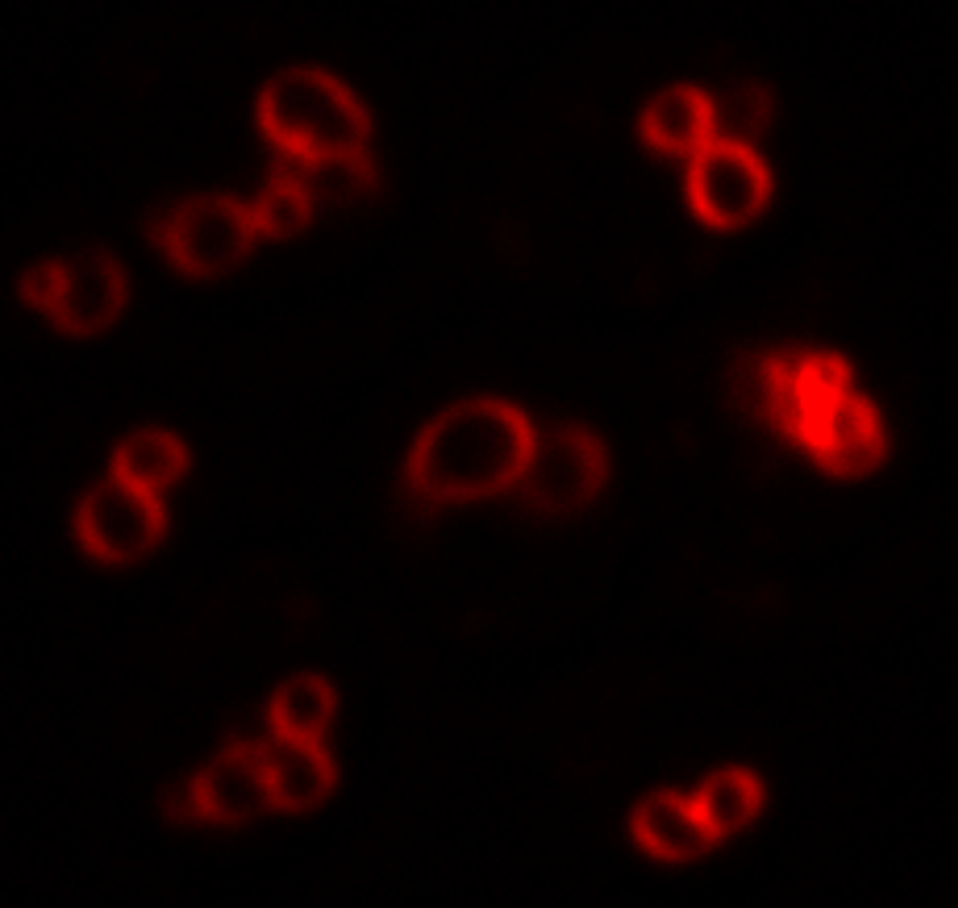

Supplement: Supplementary file 9 — Source data Fig. 8 [file 44318_2026_716_MOESM9_ESM.zip › SD Figure 8/AVG_C2-Ste13_1-1.tif]

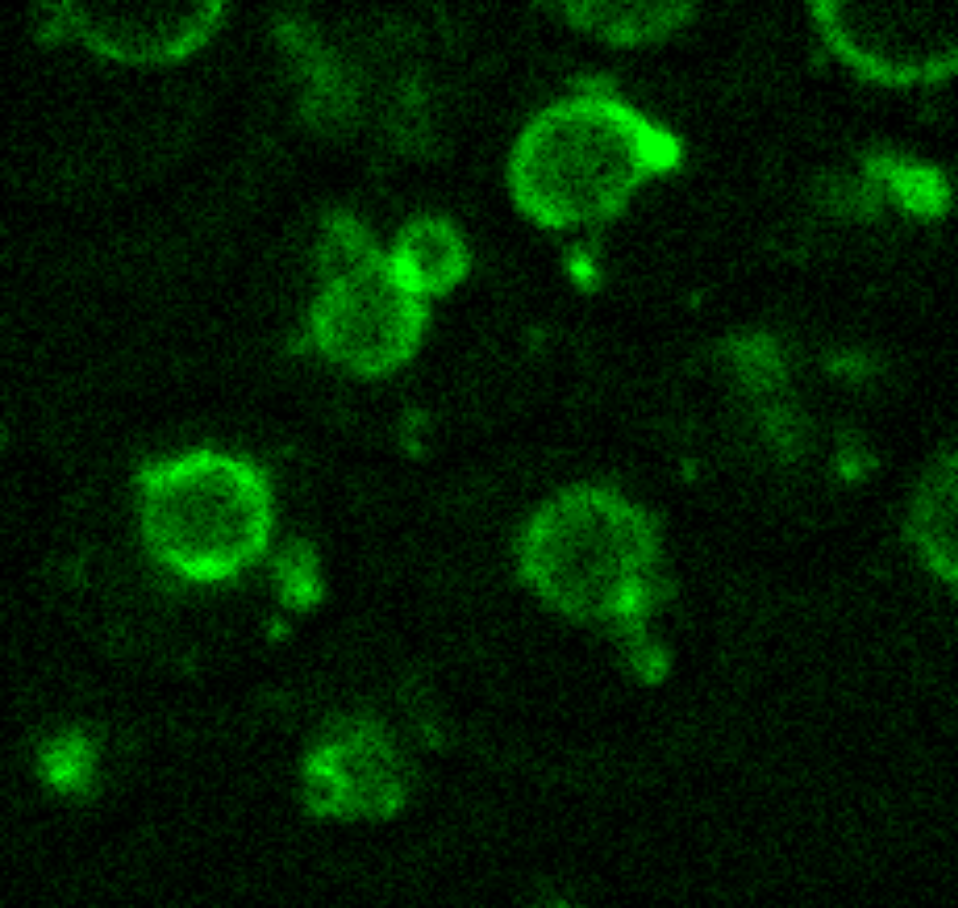

Supplement: Supplementary file 9 — Source data Fig. 8 [file 44318_2026_716_MOESM9_ESM.zip › SD Figure 8/AVG_C1-Ste13_Snx3 KO_1-1.tif]

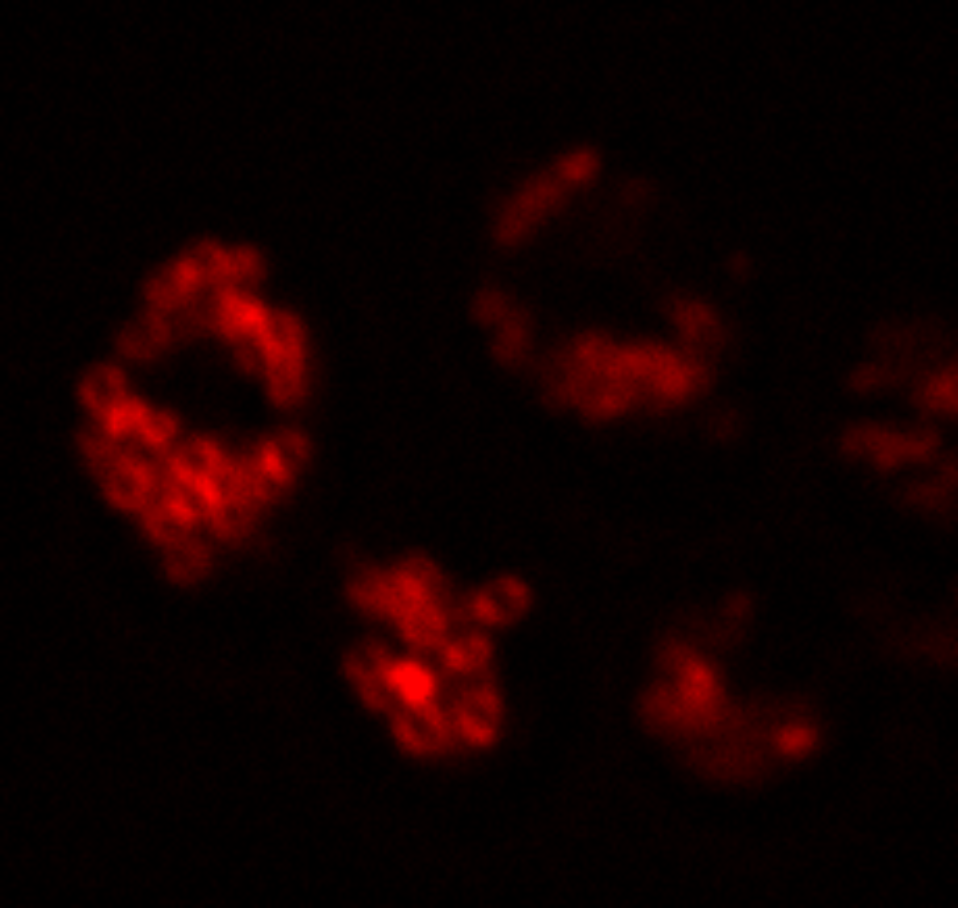

Supplement: Supplementary file 9 — Source data Fig. 8 [file 44318_2026_716_MOESM9_ESM.zip › SD Figure 8/AVG_C2-Vps17-KO_3.tif]

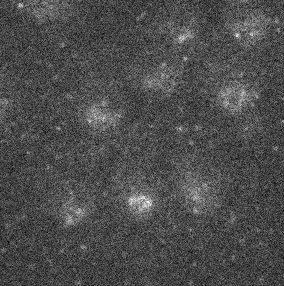

Supplement: Supplementary file 9 — Source data Fig. 8 [file 44318_2026_716_MOESM9_ESM.zip › SD Figure 8/Fig8C_vps5_KO.tif]

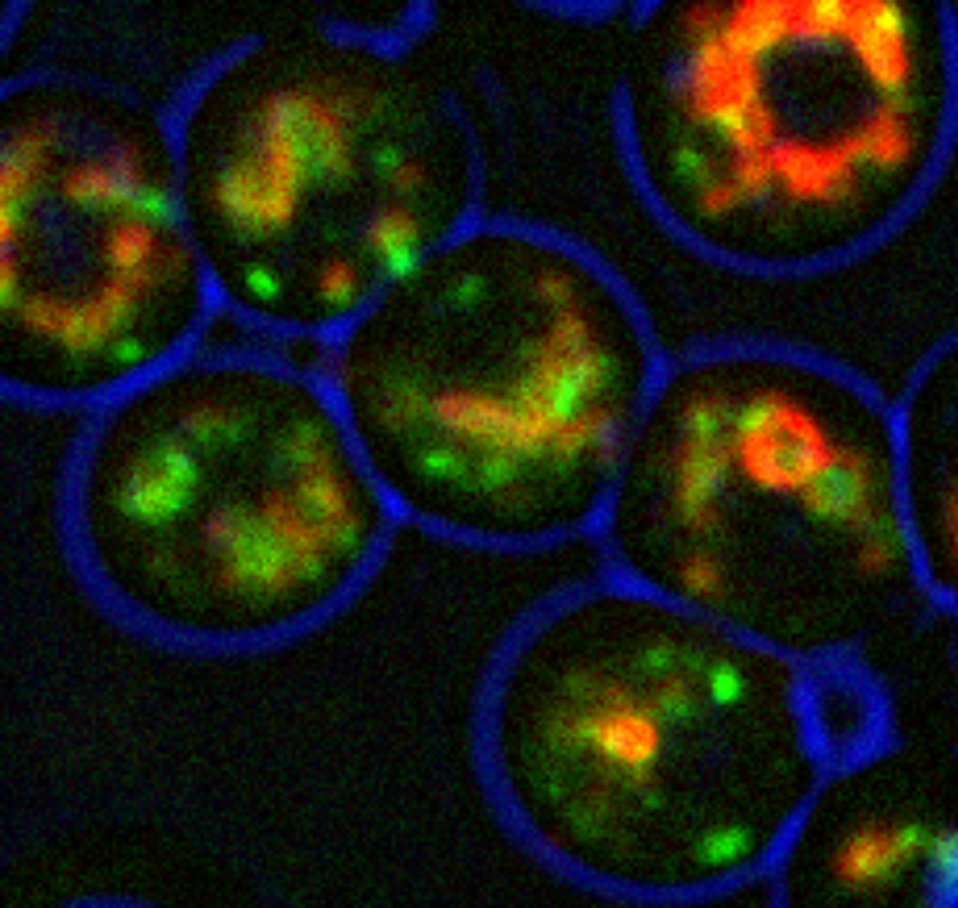

Supplement: Supplementary file 9 — Source data Fig. 8 [file 44318_2026_716_MOESM9_ESM.zip › SD Figure 8/AVG_C4-Ear_Vps17-KO002-2.tif]

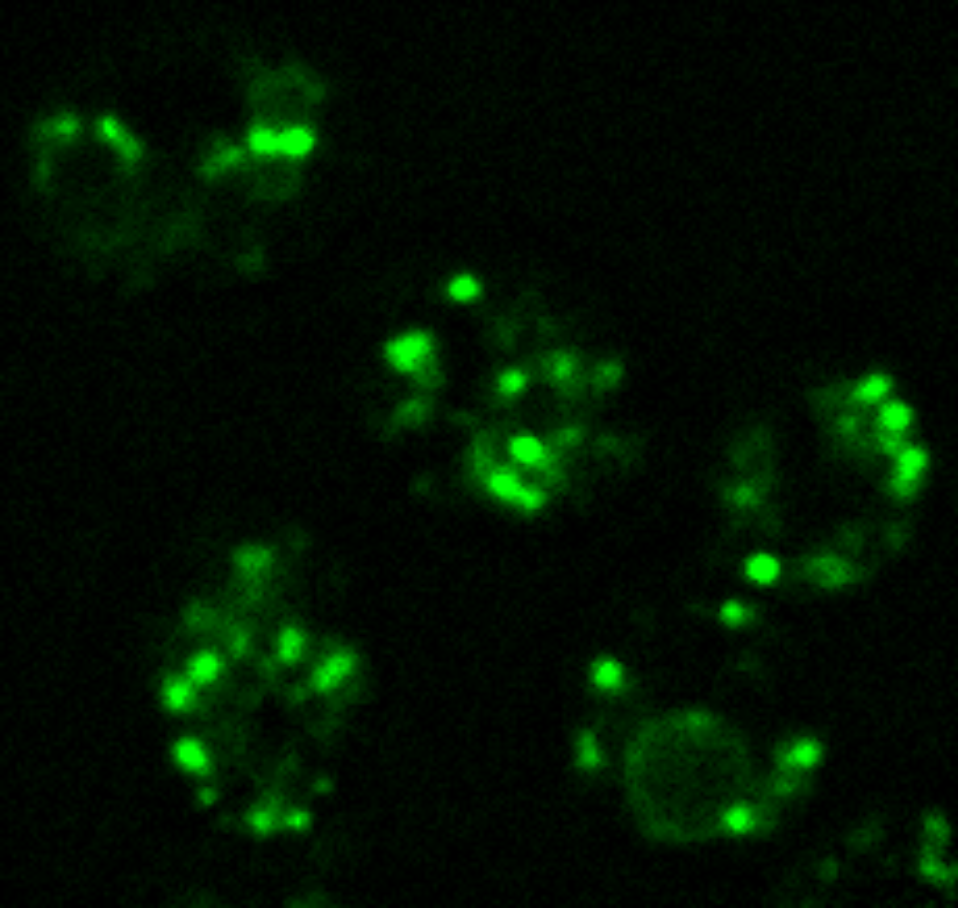

Supplement: Supplementary file 9 — Source data Fig. 8 [file 44318_2026_716_MOESM9_ESM.zip › SD Figure 8/C1-AVG_Sxn3-KO_1-1.tif]

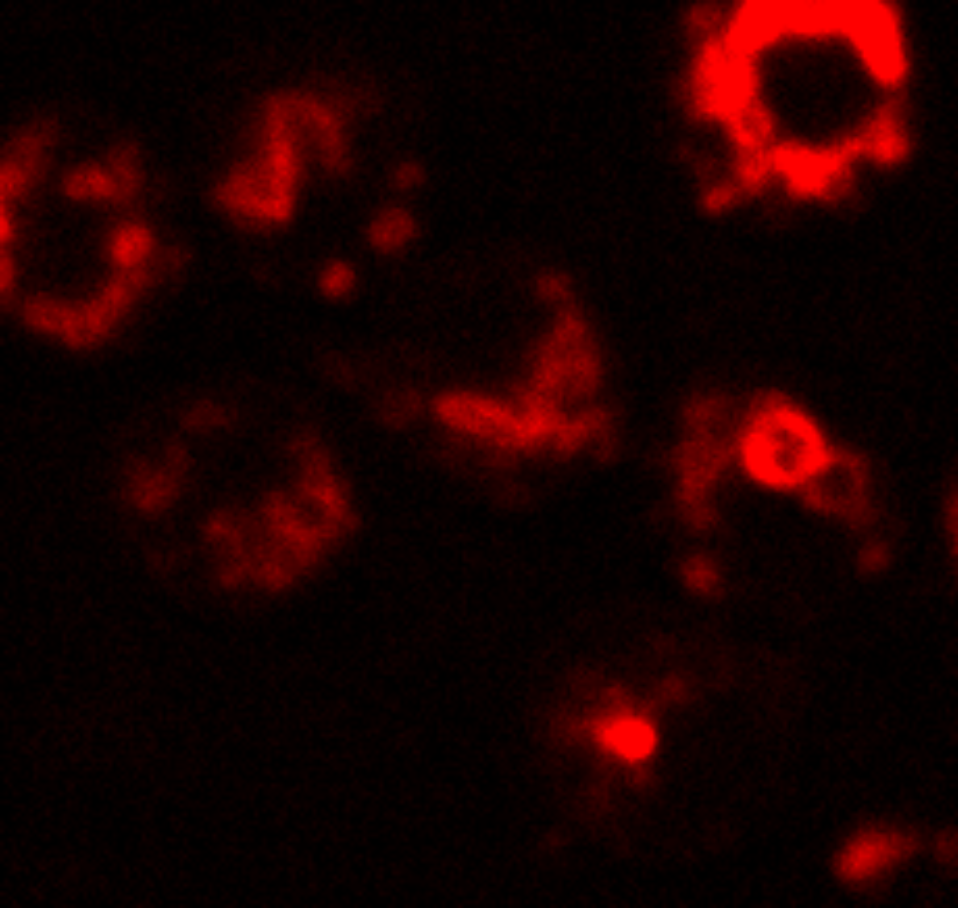

Supplement: Supplementary file 9 — Source data Fig. 8 [file 44318_2026_716_MOESM9_ESM.zip › SD Figure 8/AVG_C2-Ear_Vps17-KO002-2.tif]

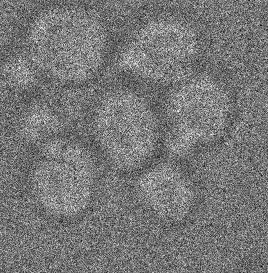

Supplement: Supplementary file 9 — Source data Fig. 8 [file 44318_2026_716_MOESM9_ESM.zip › SD Figure 8/Fig8E_ctrl.tif]

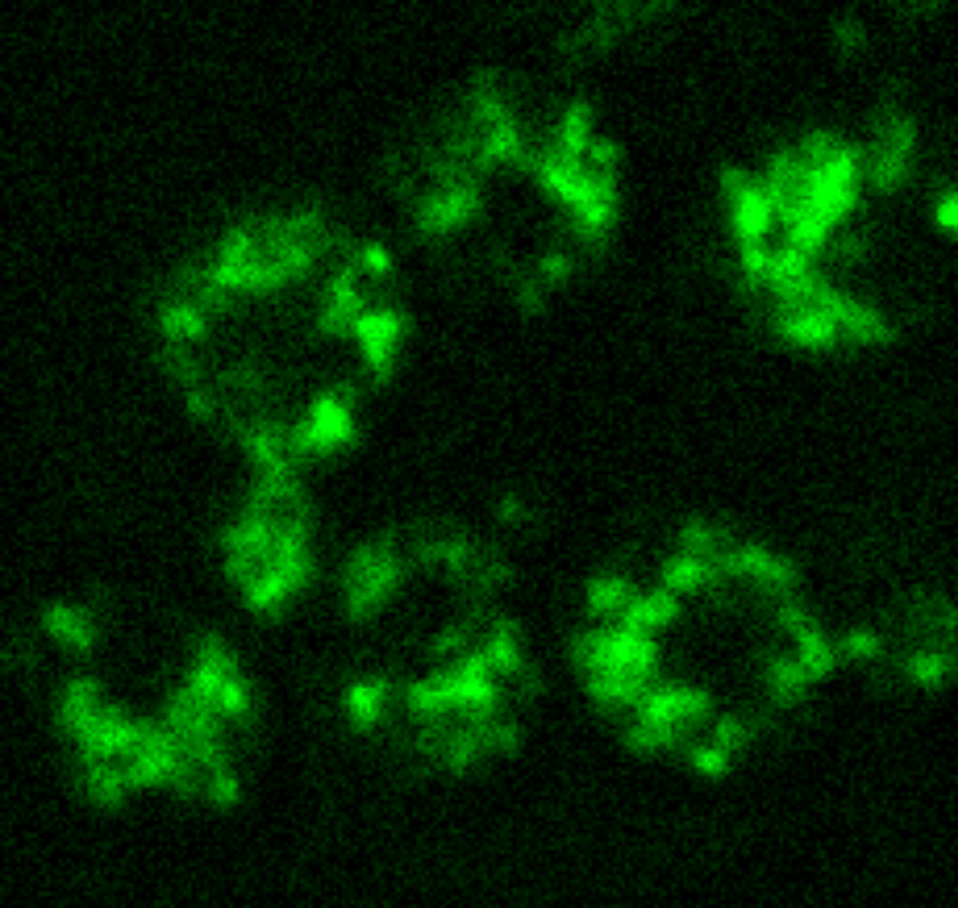

Supplement: Supplementary file 9 — Source data Fig. 8 [file 44318_2026_716_MOESM9_ESM.zip › SD Figure 8/AVG_C1-Ear_Vps5-KO001.tif]

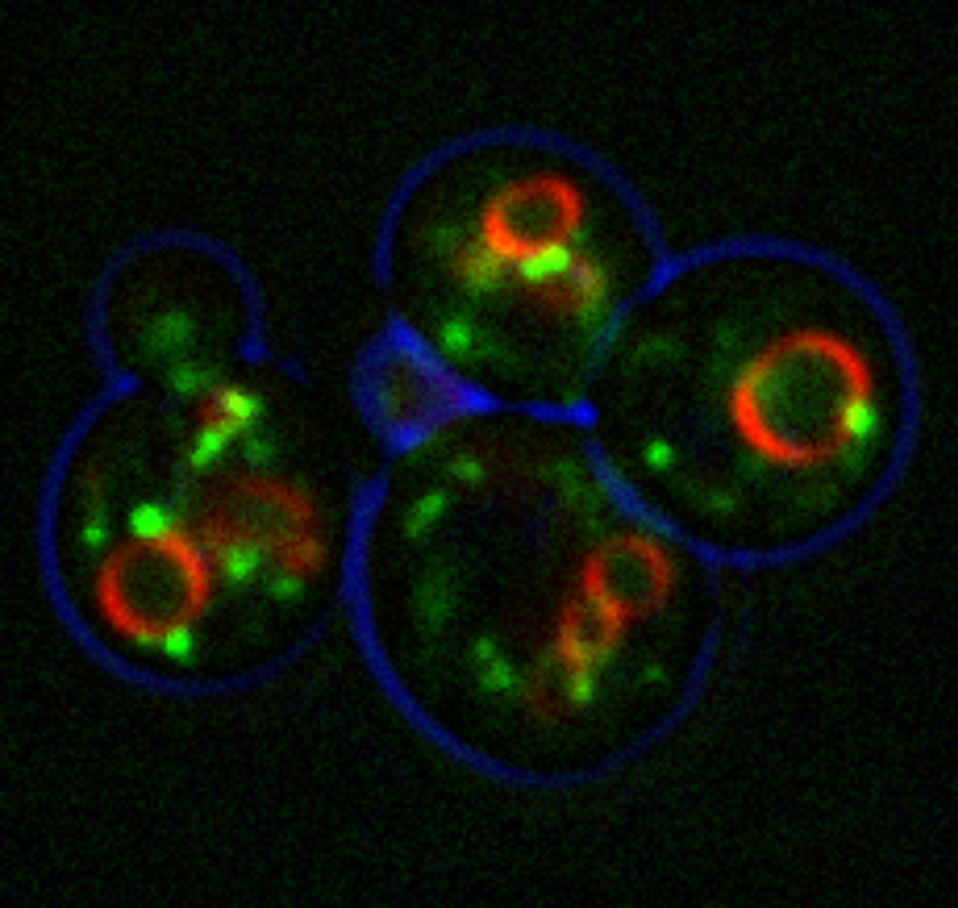

Supplement: Supplementary file 9 — Source data Fig. 8 [file 44318_2026_716_MOESM9_ESM.zip › SD Figure 8/C4-AVG_cont_1.tif]

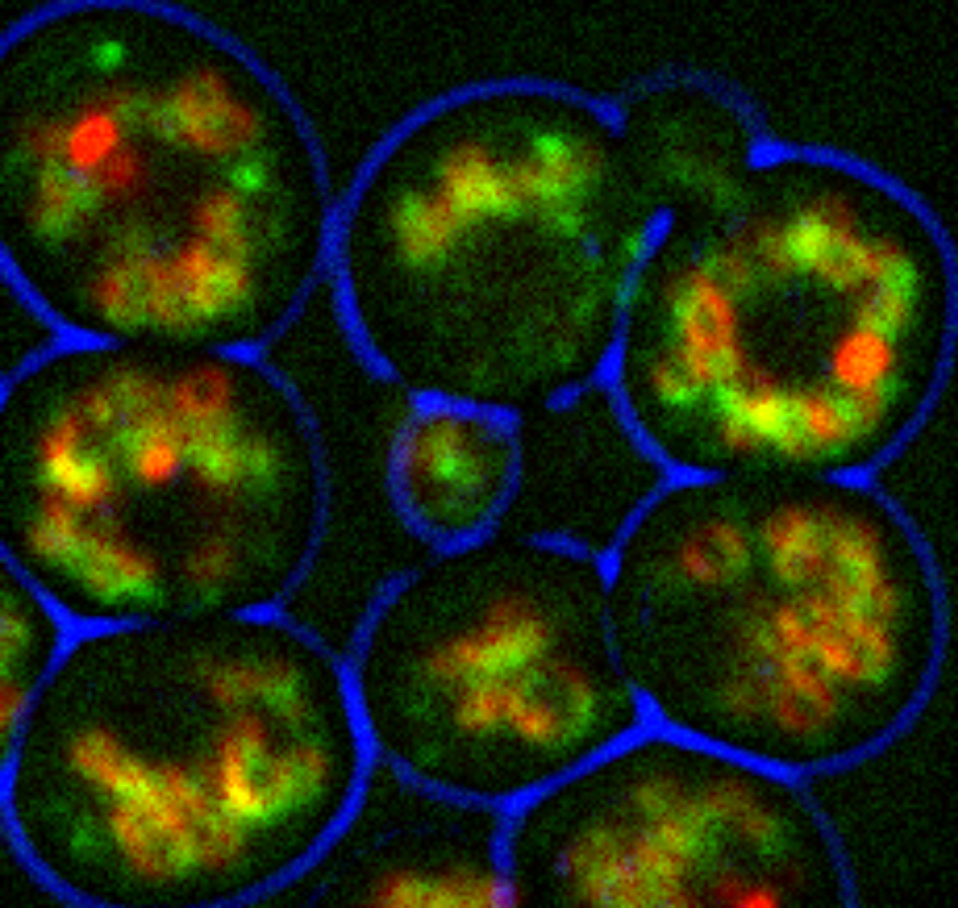

Supplement: Supplementary file 9 — Source data Fig. 8 [file 44318_2026_716_MOESM9_ESM.zip › SD Figure 8/C4-AVG_Ste13_Vps17 KO_1003-1.tif]

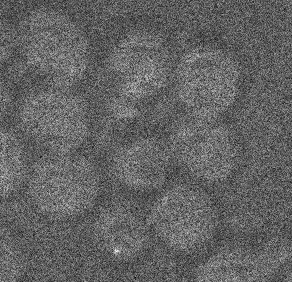

Supplement: Supplementary file 9 — Source data Fig. 8 [file 44318_2026_716_MOESM9_ESM.zip › SD Figure 8/Fig8E_vps17_KO.tif]

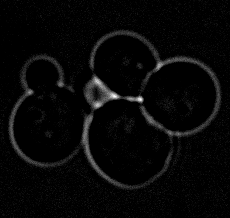

Supplement: Supplementary file 9 — Source data Fig. 8 [file 44318_2026_716_MOESM9_ESM.zip › SD Figure 8/C3-AVG_cont_1.tif]

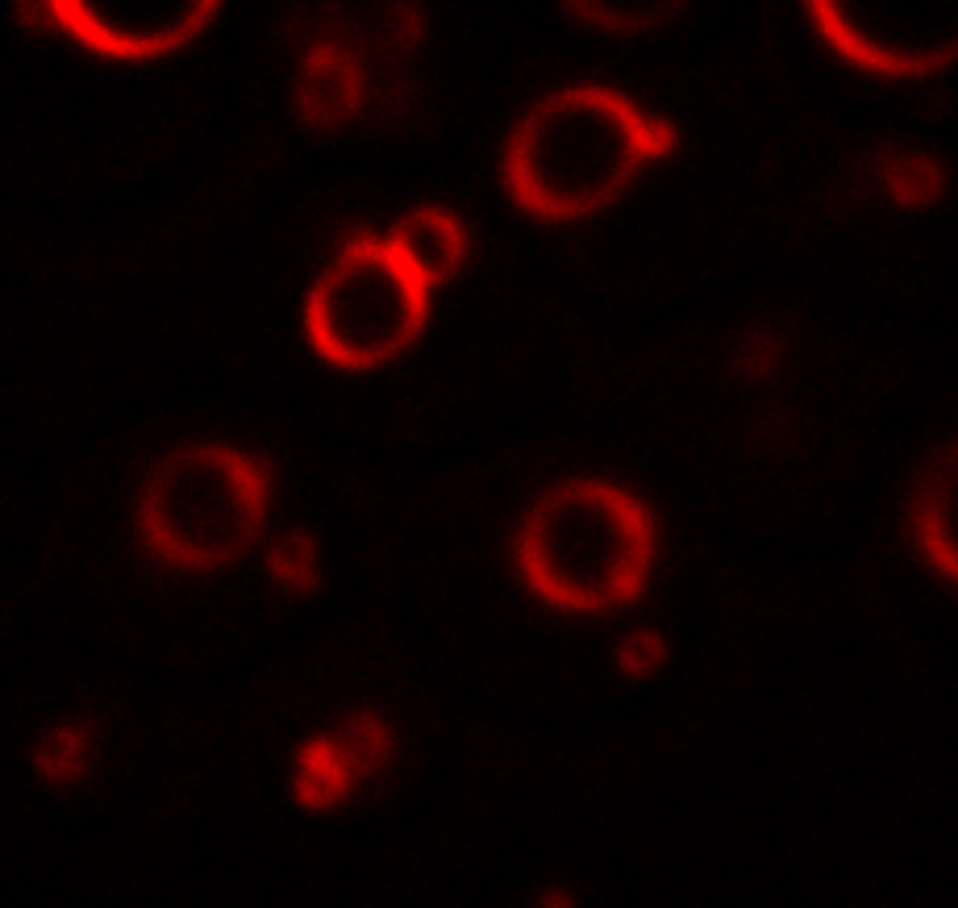

Supplement: Supplementary file 9 — Source data Fig. 8 [file 44318_2026_716_MOESM9_ESM.zip › SD Figure 8/C2-AVG_Ste13_Snx3 KO_1-1.tif]

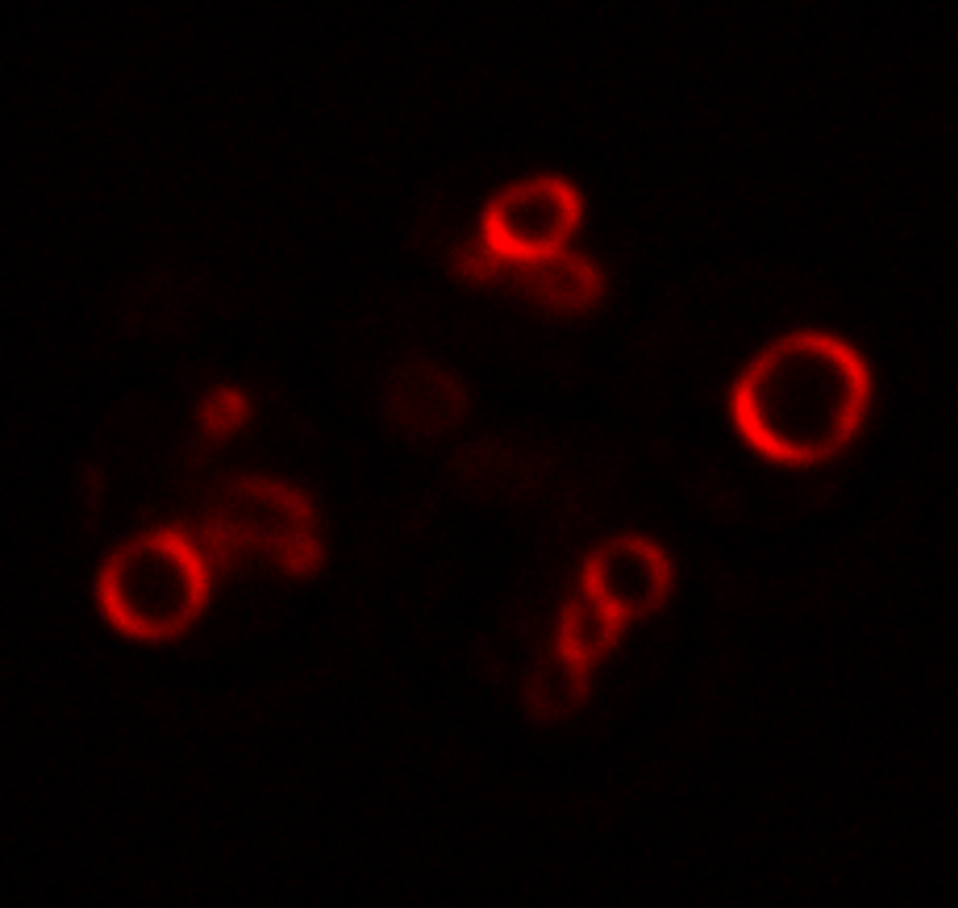

Supplement: Supplementary file 9 — Source data Fig. 8 [file 44318_2026_716_MOESM9_ESM.zip › SD Figure 8/C2-AVG_cont_1.tif]

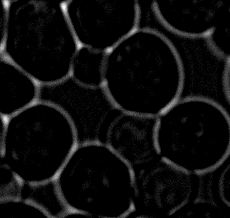

Supplement: Supplementary file 9 — Source data Fig. 8 [file 44318_2026_716_MOESM9_ESM.zip › SD Figure 8/AVG_C3-Ste13_Vps5 KO_1004-1.tif]

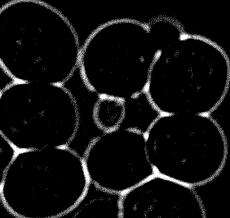

Supplement: Supplementary file 9 — Source data Fig. 8 [file 44318_2026_716_MOESM9_ESM.zip › SD Figure 8/C3-AVG_Ste13_Vps17 KO_1003-1.tif]

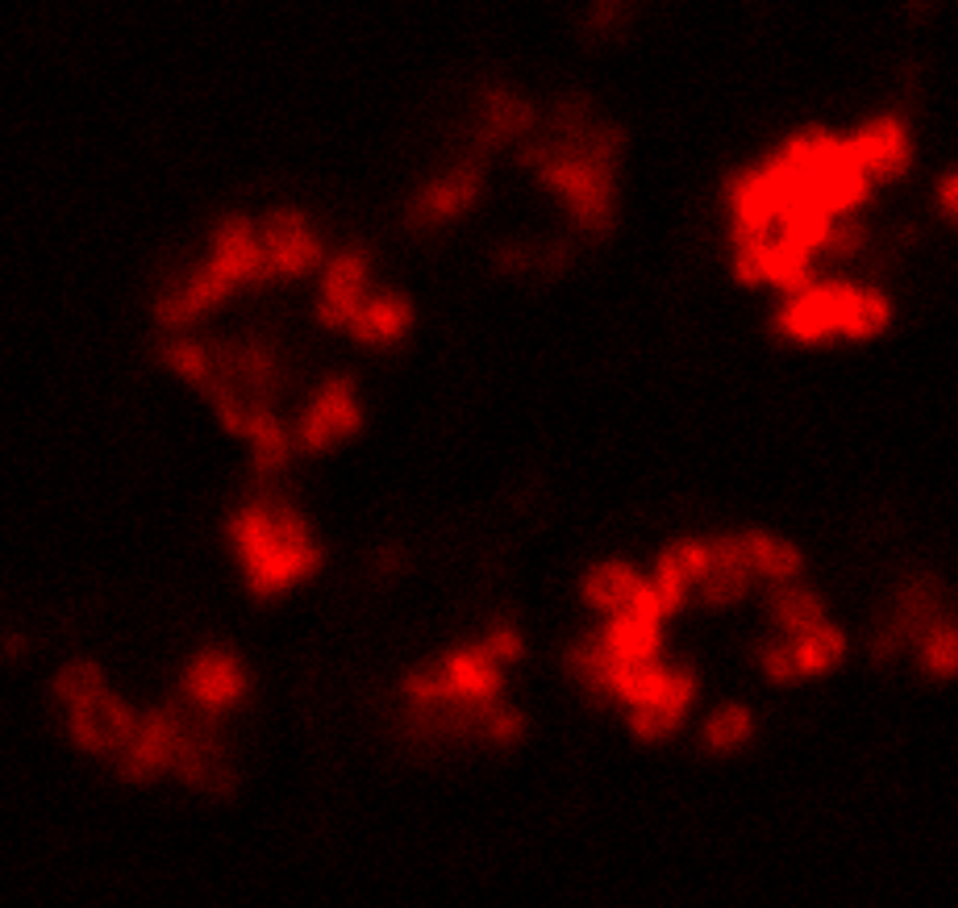

Supplement: Supplementary file 9 — Source data Fig. 8 [file 44318_2026_716_MOESM9_ESM.zip › SD Figure 8/AVG_C2-Ear_Vps5-KO001.tif]

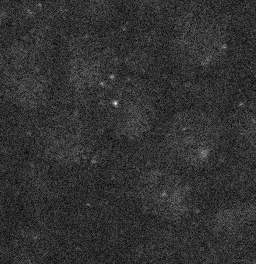

Supplement: Supplementary file 9 — Source data Fig. 8 [file 44318_2026_716_MOESM9_ESM.zip › SD Figure 8/Fig8C_vps17_KO.tif]

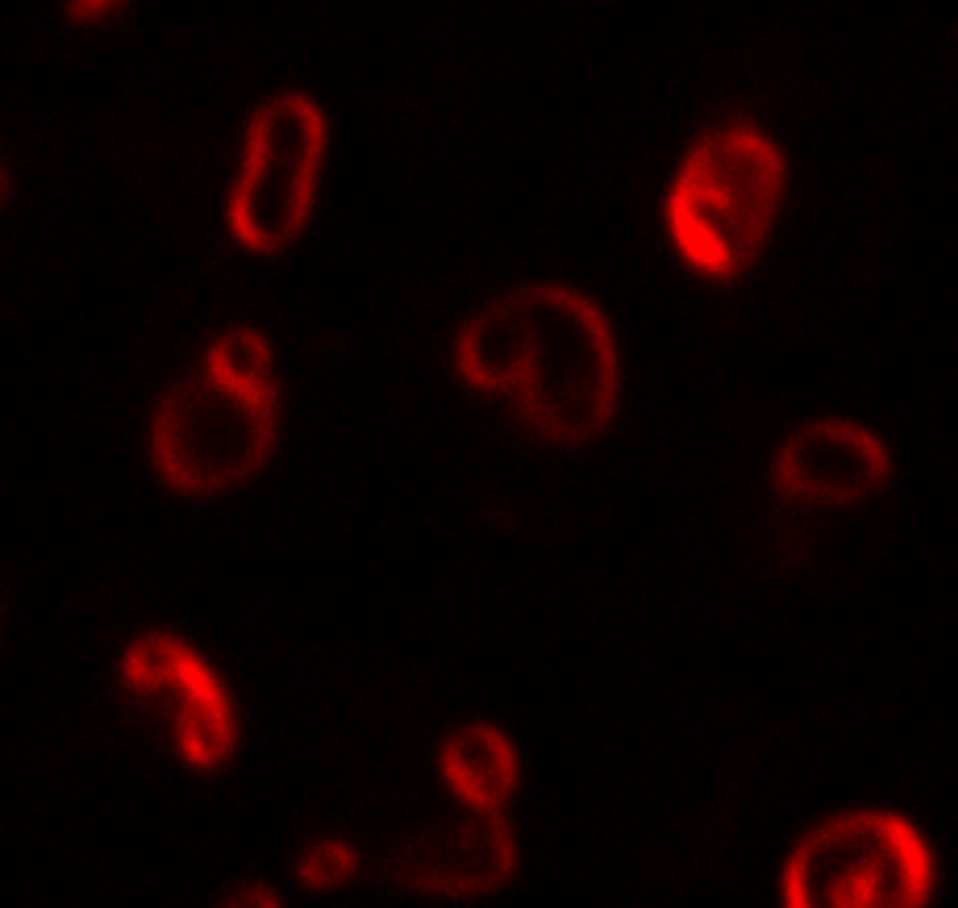

Supplement: Supplementary file 9 — Source data Fig. 8 [file 44318_2026_716_MOESM9_ESM.zip › SD Figure 8/AVG_C2-Ear1-1.tif]

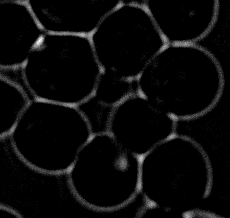

Supplement: Supplementary file 9 — Source data Fig. 8 [file 44318_2026_716_MOESM9_ESM.zip › SD Figure 8/AVG_C3-Ear_Snx3-KO001-1.tif]

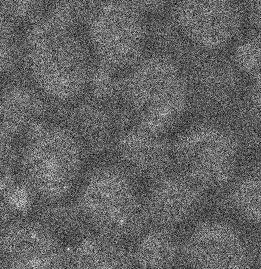

Supplement: Supplementary file 9 — Source data Fig. 8 [file 44318_2026_716_MOESM9_ESM.zip › SD Figure 8/Fig8E_vps5_KO.tif]

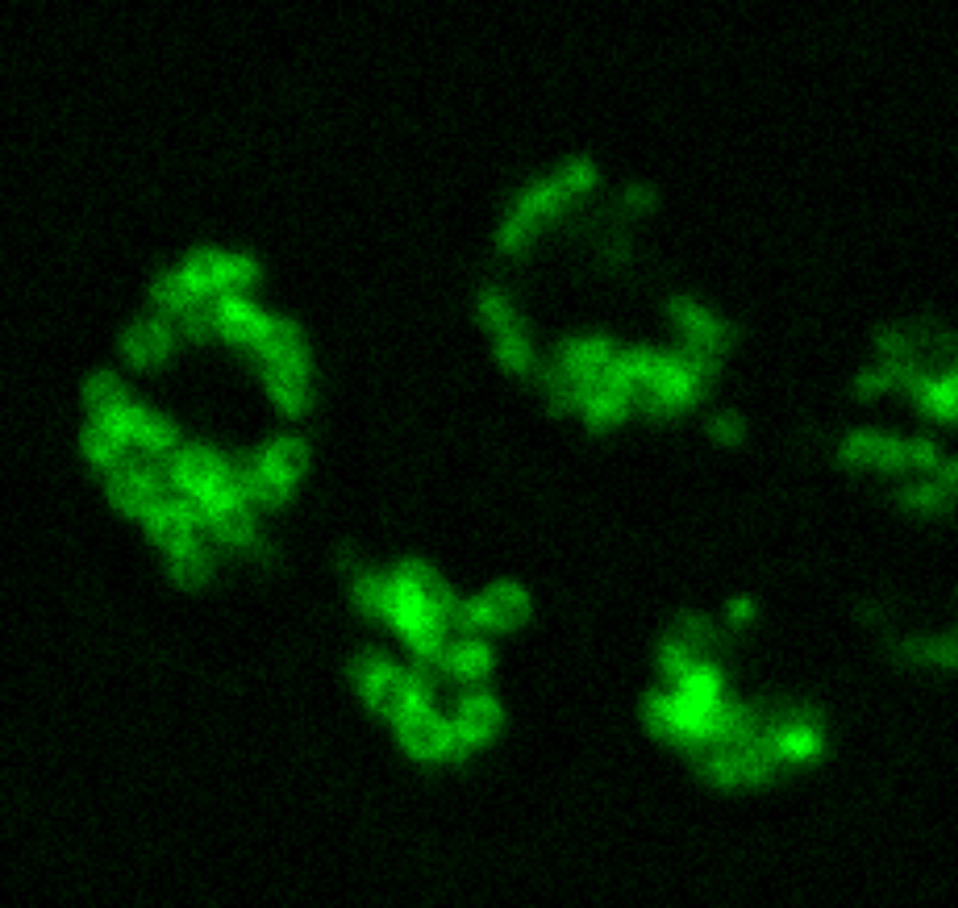

Supplement: Supplementary file 9 — Source data Fig. 8 [file 44318_2026_716_MOESM9_ESM.zip › SD Figure 8/AVG_C1-Vps17-KO_3.tif]

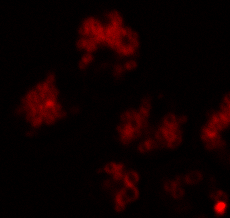

Supplement: Supplementary file 10 — Figures EV Source Data [file 44318_2026_716_MOESM10_ESM.zip › SD Figures EV/C2-AVG_Vps17KO-Pep12_3-1.tif]

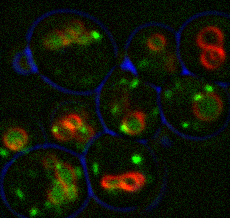

Supplement: Supplementary file 10 — Figures EV Source Data [file 44318_2026_716_MOESM10_ESM.zip › SD Figures EV/C4-AVG_Kex2_1.tif]

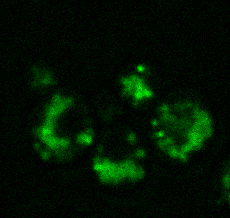

Supplement: Supplementary file 10 — Figures EV Source Data [file 44318_2026_716_MOESM10_ESM.zip › SD Figures EV/C1-AVG_Kex2_vps17 KO_1004-1.tif]

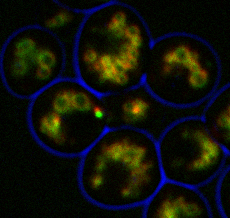

Supplement: Supplementary file 10 — Figures EV Source Data [file 44318_2026_716_MOESM10_ESM.zip › SD Figures EV/C4-AVG_Vps5KO-Pep12_2-1.tif]

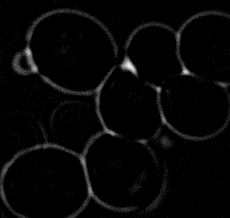

Supplement: Supplementary file 10 — Figures EV Source Data [file 44318_2026_716_MOESM10_ESM.zip › SD Figures EV/C3-AVG_Kex2_1.tif]

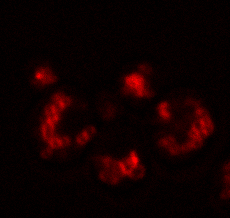

Supplement: Supplementary file 10 — Figures EV Source Data [file 44318_2026_716_MOESM10_ESM.zip › SD Figures EV/C2-AVG_Kex2_vps17 KO_1004-1.tif]

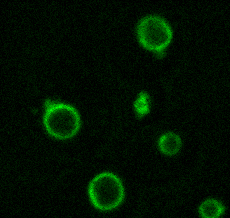

Supplement: Supplementary file 10 — Figures EV Source Data [file 44318_2026_716_MOESM10_ESM.zip › SD Figures EV/C1-AVG_Snx3KO-Pep12_3-1.tif]

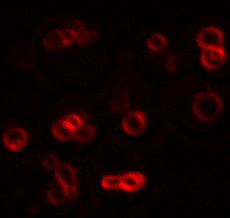

Supplement: Supplementary file 10 — Figures EV Source Data [file 44318_2026_716_MOESM10_ESM.zip › SD Figures EV/C2-AVG_Kex2_1.tif]

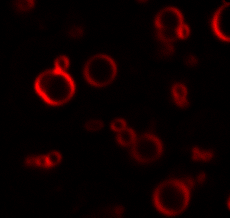

Supplement: Supplementary file 10 — Figures EV Source Data [file 44318_2026_716_MOESM10_ESM.zip › SD Figures EV/C2-AVG_Kex2_snx3 KO_1001-1.tif]

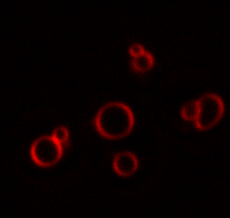

Supplement: Supplementary file 10 — Figures EV Source Data [file 44318_2026_716_MOESM10_ESM.zip › SD Figures EV/C2-AVG_Cont_4-1.tif]

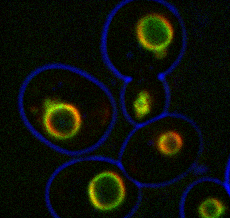

Supplement: Supplementary file 10 — Figures EV Source Data [file 44318_2026_716_MOESM10_ESM.zip › SD Figures EV/C4-AVG_Snx3KO-Pep12_3-1.tif]

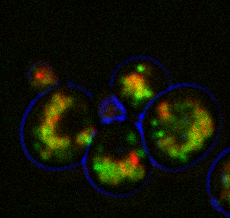

Supplement: Supplementary file 10 — Figures EV Source Data [file 44318_2026_716_MOESM10_ESM.zip › SD Figures EV/C4-AVG_Kex2_vps17 KO_1004-1.tif]

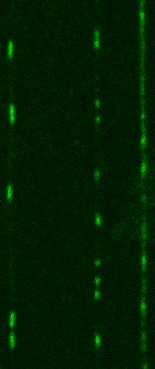

Supplement: Supplementary file 10 — Figures EV Source Data [file 44318_2026_716_MOESM10_ESM.zip › SD Figures EV/AVG_C3-28-10-24_Vps10-546+sbg_cc002.tif]

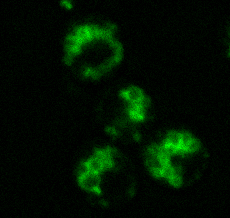

Supplement: Supplementary file 10 — Figures EV Source Data [file 44318_2026_716_MOESM10_ESM.zip › SD Figures EV/C1-AVG_Kex2_vps5KO_1004-1.tif]

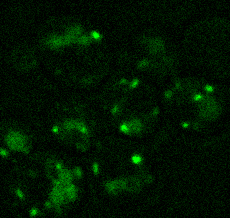

Supplement: Supplementary file 10 — Figures EV Source Data [file 44318_2026_716_MOESM10_ESM.zip › SD Figures EV/C1-AVG_Kex2_1.tif]

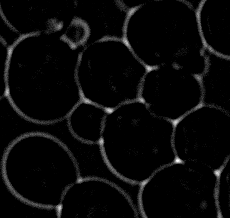

Supplement: Supplementary file 10 — Figures EV Source Data [file 44318_2026_716_MOESM10_ESM.zip › SD Figures EV/C3-AVG_Kex2_snx3 KO_1001-1.tif]

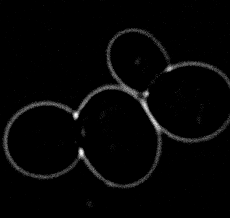

Supplement: Supplementary file 10 — Figures EV Source Data [file 44318_2026_716_MOESM10_ESM.zip › SD Figures EV/C3-AVG_Cont_4-1.tif]

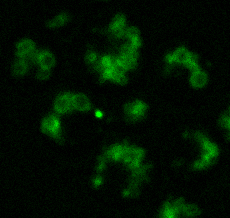

Supplement: Supplementary file 10 — Figures EV Source Data [file 44318_2026_716_MOESM10_ESM.zip › SD Figures EV/C1-AVG_Vps5KO-Pep12_2-1.tif]

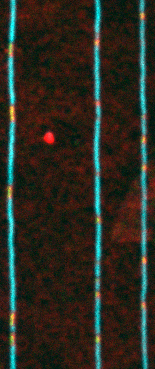

Supplement: Supplementary file 10 — Figures EV Source Data [file 44318_2026_716_MOESM10_ESM.zip › SD Figures EV/AVG_C1-28-10-24_Vps10-546+sbg_cc002.tif]

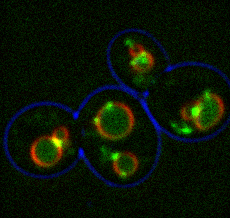

Supplement: Supplementary file 10 — Figures EV Source Data [file 44318_2026_716_MOESM10_ESM.zip › SD Figures EV/C4-AVG_Cont_4-1.tif]

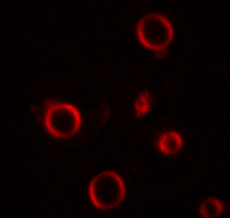

Supplement: Supplementary file 10 — Figures EV Source Data [file 44318_2026_716_MOESM10_ESM.zip › SD Figures EV/C2-AVG_Snx3KO-Pep12_3-1.tif]

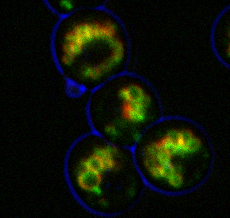

Supplement: Supplementary file 10 — Figures EV Source Data [file 44318_2026_716_MOESM10_ESM.zip › SD Figures EV/C4-AVG_Kex2_vps5KO_1004-1.tif]

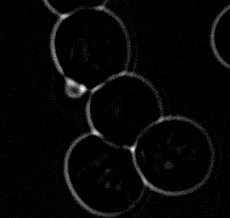

Supplement: Supplementary file 10 — Figures EV Source Data [file 44318_2026_716_MOESM10_ESM.zip › SD Figures EV/C3-AVG_Kex2_vps5KO_1004-1.tif]

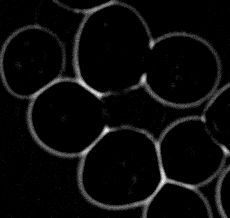

Supplement: Supplementary file 10 — Figures EV Source Data [file 44318_2026_716_MOESM10_ESM.zip › SD Figures EV/C3-AVG_Vps5KO-Pep12_2-1.tif]

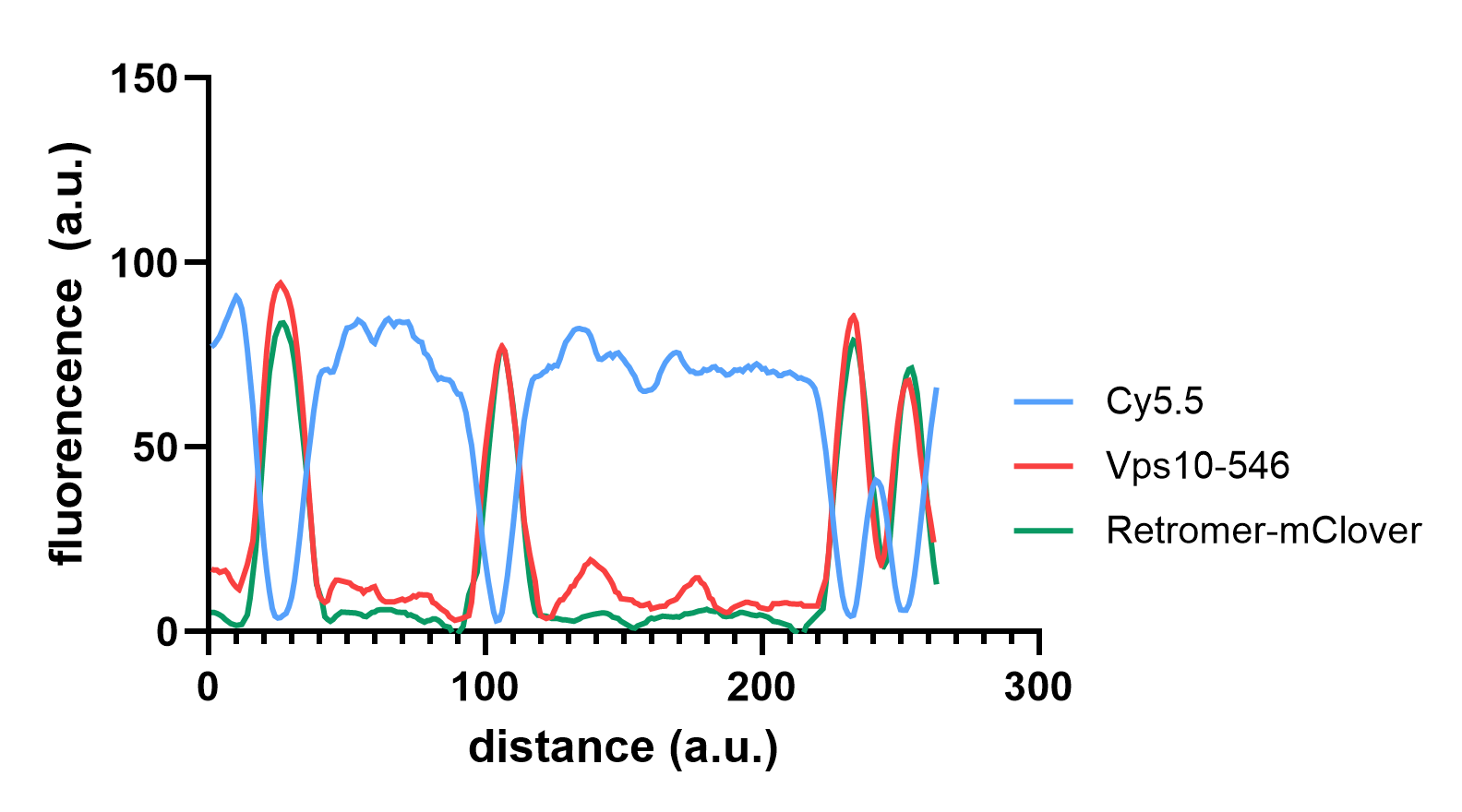

Supplement: Supplementary file 10 — Figures EV Source Data [file 44318_2026_716_MOESM10_ESM.zip › SD Figures EV/Normalize of Vps10-546+SNX-BAR+CSC-mClover.tif]

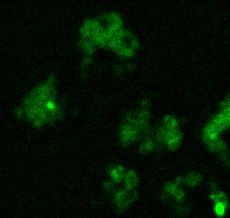

Supplement: Supplementary file 10 — Figures EV Source Data [file 44318_2026_716_MOESM10_ESM.zip › SD Figures EV/C1-AVG_Vps17KO-Pep12_3-1.tif]

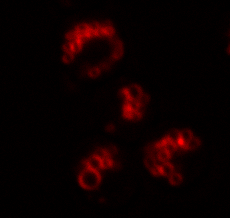

Supplement: Supplementary file 10 — Figures EV Source Data [file 44318_2026_716_MOESM10_ESM.zip › SD Figures EV/C2-AVG_Kex2_vps5KO_1004-1.tif]

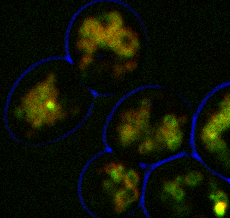

Supplement: Supplementary file 10 — Figures EV Source Data [file 44318_2026_716_MOESM10_ESM.zip › SD Figures EV/C4-AVG_Vps17KO-Pep12_3-1.tif]

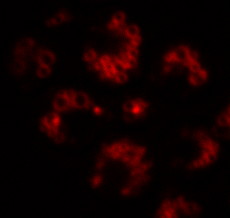

Supplement: Supplementary file 10 — Figures EV Source Data [file 44318_2026_716_MOESM10_ESM.zip › SD Figures EV/C2-AVG_Vps5KO-Pep12_2-1.tif]

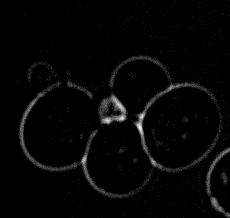

Supplement: Supplementary file 10 — Figures EV Source Data [file 44318_2026_716_MOESM10_ESM.zip › SD Figures EV/C3-AVG_Kex2_vps17 KO_1004-1.tif]

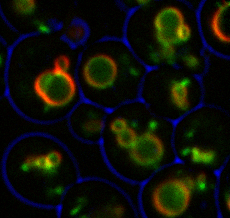

Supplement: Supplementary file 10 — Figures EV Source Data [file 44318_2026_716_MOESM10_ESM.zip › SD Figures EV/C4-AVG_Kex2_snx3 KO_1001-1.tif]

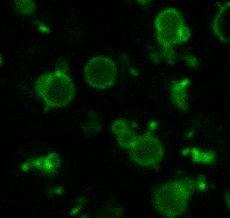

Supplement: Supplementary file 10 — Figures EV Source Data [file 44318_2026_716_MOESM10_ESM.zip › SD Figures EV/C1-AVG_Kex2_snx3 KO_1001-1.tif]

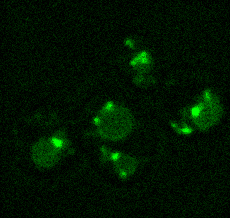

Supplement: Supplementary file 10 — Figures EV Source Data [file 44318_2026_716_MOESM10_ESM.zip › SD Figures EV/C1-AVG_Cont_4-1.tif]

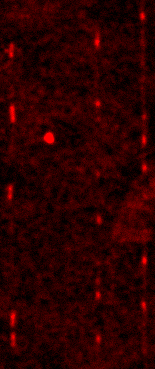

Supplement: Supplementary file 10 — Figures EV Source Data [file 44318_2026_716_MOESM10_ESM.zip › SD Figures EV/AVG_C2-28-10-24_Vps10-546+sbg_cc002.tif]

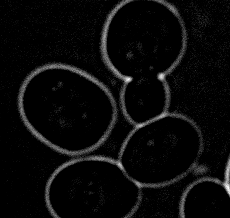

Supplement: Supplementary file 10 — Figures EV Source Data [file 44318_2026_716_MOESM10_ESM.zip › SD Figures EV/C3-AVG_Snx3KO-Pep12_3-1.tif]

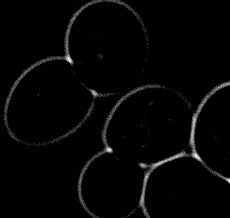

Supplement: Supplementary file 10 — Figures EV Source Data [file 44318_2026_716_MOESM10_ESM.zip › SD Figures EV/C3-AVG_Vps17KO-Pep12_3-1.tif]
