# Supplementary material for: Hybrid endosomal coats contain different classes of sorting nexins
Source: EMBO J. 2026 Feb 16;45(7):2278–305. doi: 10.1038/s44318-026-00716-0 (PMC13043683; doi:10.1038/s44318-026-00716-0)
Supplement: Supplementary file 11 — Expanded View Figures [file 44318_2026_716_MOESM11_ESM.pdf]

Expanded View Figures

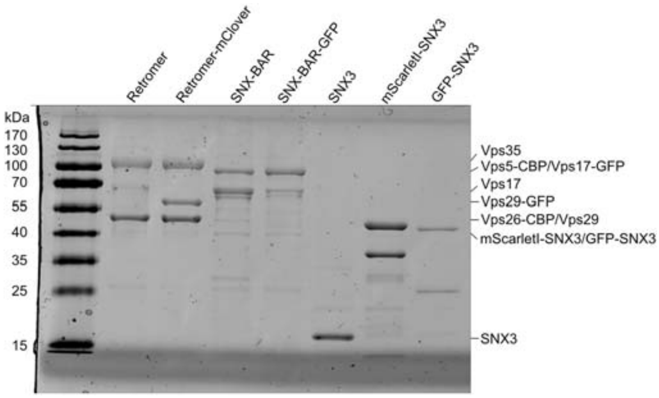

Figure EV1. Protein preparations.

Coomassie-stained SDS-PAGE gel of the protein preparations used in the in vitro experiments.

 © The Author(s)

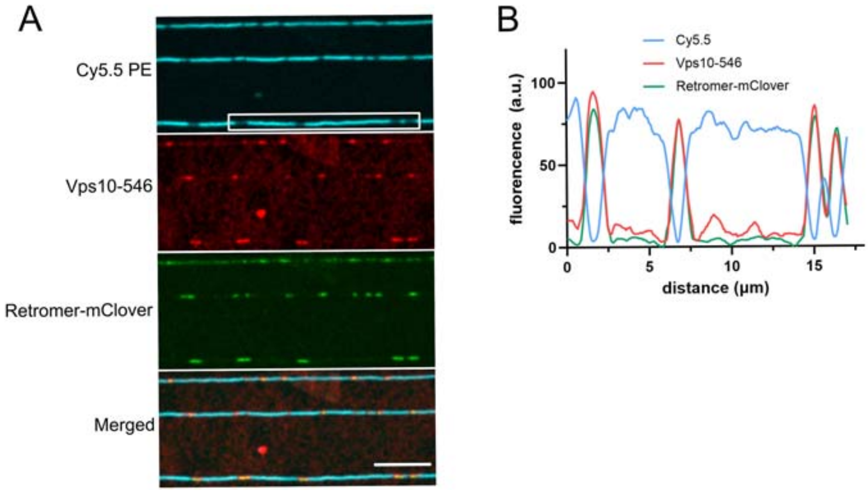

Figure EV2. Vps10 cargo peptide concentration by SNX-BAR-Retromer coats.

(**A**) SMTubes were formed and pre-incubated with 5 μM fluorescently labelled cargo peptide Vps10-546 for 10 mins, followed by addition of 25 nM SNX-BAR and 25 nM Retromer^{mClover} until coat formation became apparent (2–3 min). After a brief wash with phosphate-buffered saline (PBS), tubes were imaged by spinning disc confocal microscopy. Scale bar: 2 μm. (**B**) Line scan of the region boxed in A.

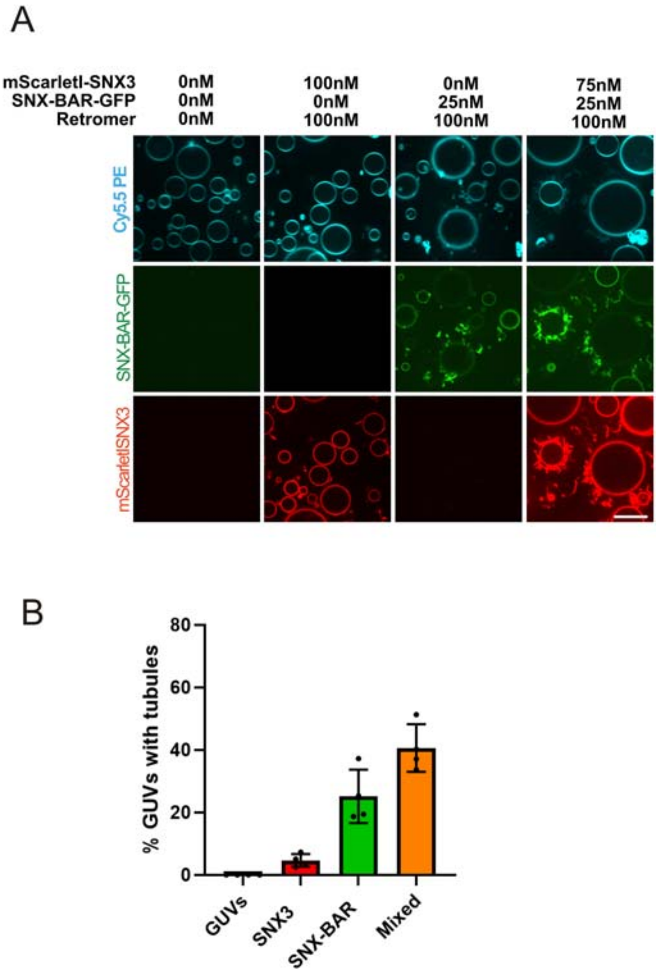

Figure EV3. Tubulation of GUVs by hybrid coats in the presence of SNX-BAR and Snx3 cargos.

(**A**) GUVs labelled with Cy5.5-PE were incubated with the indicated concentrations of Retromer, ^{mScarletI}Snx3 and SNX-BAR^{GFP}, and with 5 µM each of Ear1 and Vps10 peptide. After 1 h of incubation, the GUVs were imaged by confocal microscopy. (**B**) Quantification of the percentage of GUVs with tubules shown in (**A**). The mean and standard deviation from 4 independent experiments were determined. Total number of scored items: GUVs only ($n = 108$); Snx3/Retromer ($n = 14$); SNX-BAR/Retromer ($n = 190$); Snx3/SNX-BAR/Retromer ($n = 167$). Scale bar: 5 µm.

 © The Author(s)

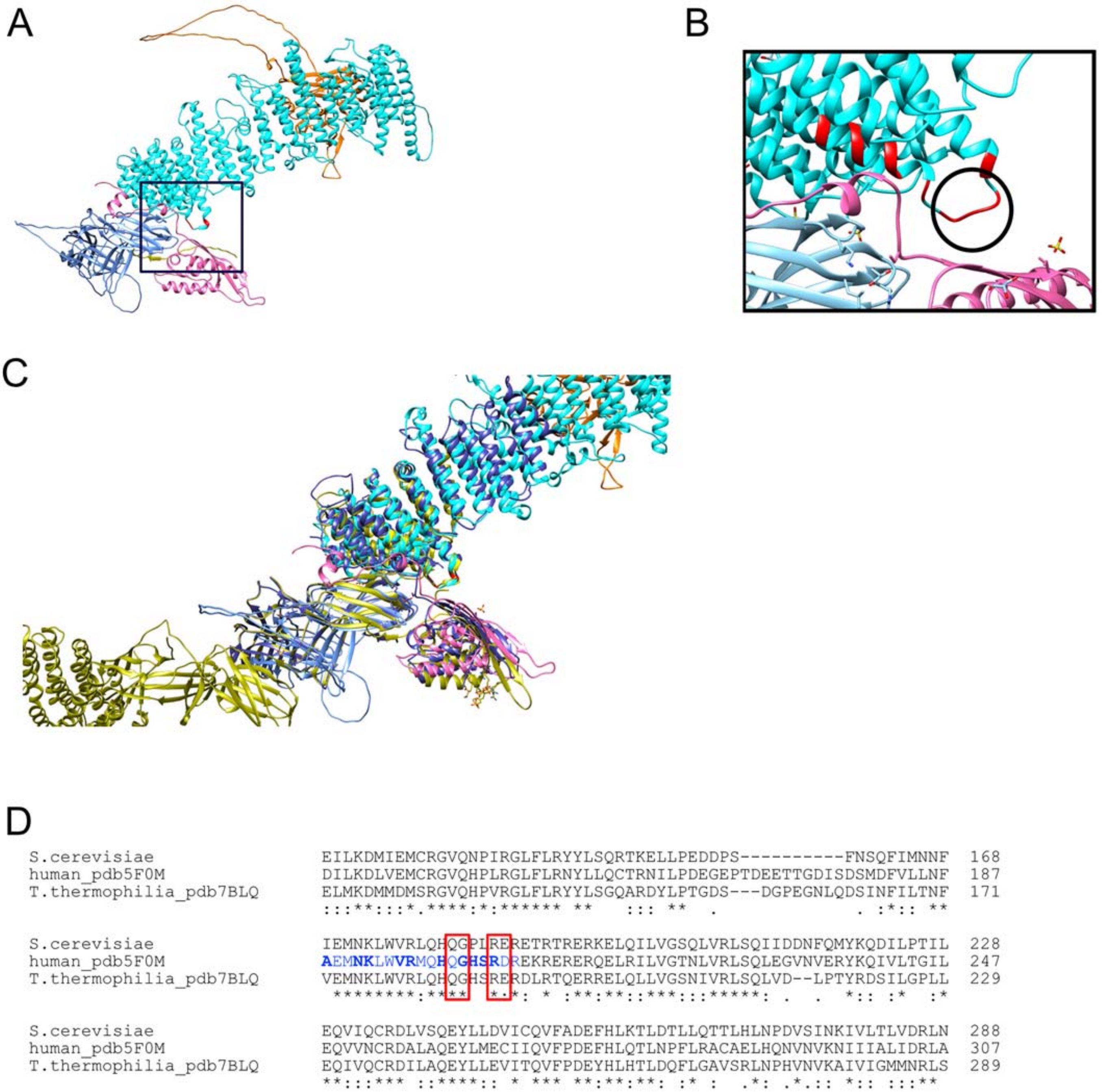

Figure EV4. Structural context of the Vps35^{QGRE} mutant.

(**A**) Alphafold 3 model of the S. cerevisiae Retromer in the presence of the Ear1 cargo peptide and Snx3. Snx3: pink; Vps26: blue; Vps35: cyan; Ear1 peptide: yellow; Substituted QGRE residues: red (**B**). Region of the human Retromer-Snx3 crystal structure (PDB 5F0M) from Hierro et al that corresponds to the boxed region of the Alphafold model in A The residues predicted by the PDB-PISA software (https://www.ebi.ac.uk/pdbe/pisa/) to participate in Vps35-Snx3 interface are shown in red. The circle highlights the substituted residues in Vps35^{QGRE} (**C**). Overlay of the Retromer-Snx3 structures from (H). sapiens (PDB 5F0M; purple) and T. thermophila (PDB 7BLQ) with the Alphafold model of S. cerevisiae (pink: Snx3, cyan: Vps35, blue: Vps26). (**D**) Alignment of the Vps35 sequences from *H. sapiens*, *T. thermophila* and *S. cerevisiae*. Residues substituted in Vps35^{QGRE} are boxed in red. The interface predicted by PISA is marked in bold type.

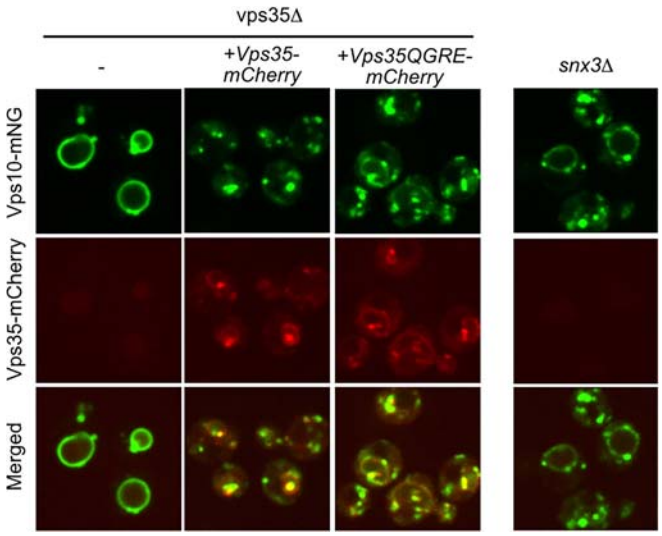

Figure EV5. Mislocalization of Vps10 in *vps35*^{QGRE} cells.

Logarithmically growing *vps35Δ* cells expressing genomically tagged Vps10^{mNG} were transformed with integrative plasmids expressing Vps35^{mCherry} (WT), Vps35$^{QGRE\text{-}mCherry}$, or nothing. The cells were logarithmically grown overnight and analysed by spinning disc microscopy. Scale bar: 5 µm.

 © The Author(s)

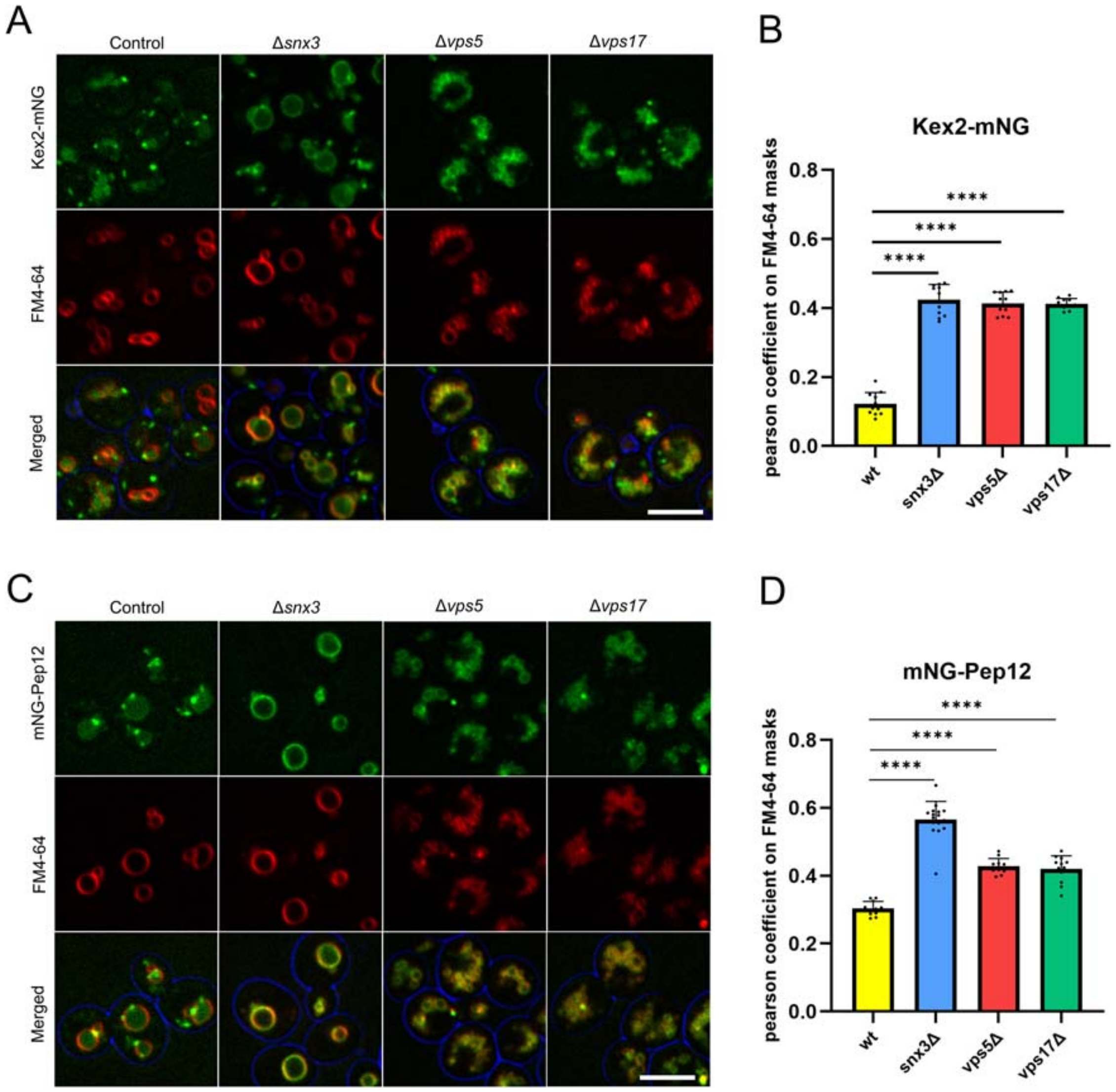

Figure EV6. Mutual dependence of Snx3 and SNX-BARs for cargo recycling.

(**A**) Kex2 distribution. Logarithmically growing cells expressing Kex2^{mNG} in wild-type, *Snx3*Δ, *vps5*Δ, and *vps17*Δ background were stained with FM4-64 and analysed by confocal microscopy. Average intensity projections of confocal z-stacks taken at a z-interval of 0.3 µm are shown. (**B**) Quantification of data from (**A**). Pearson correlation coefficients values were calculated using a Python-based app on FM4-64 masks, defining a zone close to the red signal around the vacuolar membrane. At least 5 confocal planes per experiment with 20–30 cells each from two independent experiments were analysed. Data are presented as mean values +/− standard deviation. Asterisks indicate statistical significance using two-tailed unpaired *t* tests. ****$P < 0.0001$. See also Fig. EV7C for biological replicate variability. (**C**) Same experiment as in (**A**), but with cells expressing ^{mNG}Pep12 instead of Kex2^{mNG}. (**D**) Quantification of data from (**C**), performed as in (**B**). Asterisks indicate statistical significance using two-tailed unpaired *t* tests. ****$P < 0.0001$.

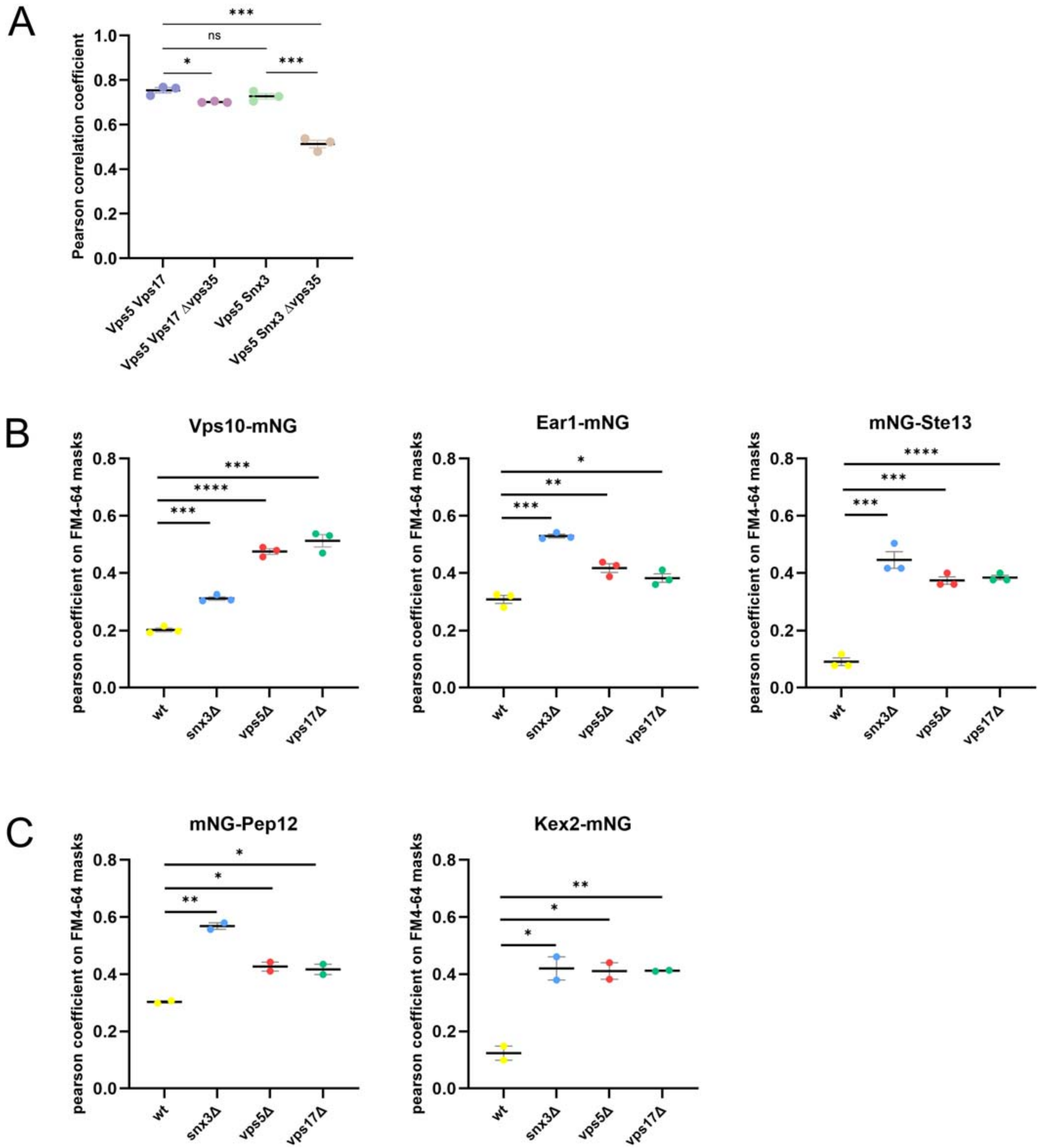

Figure EV7. Experiment-to-experiment variation in microscopic in vivo analyses.

(**A**) The quantification data from Fig. 7B was pooled per biological replicate to visualise experiment to experiment variation. (**B**) The quantification data from Fig. 8B,D,F was pooled per biological replicate to visualise experiment to experiment variation. (**C**) The quantification data from Fig. EV6B,D was pooled per biological replicate to visualise experiment to experiment variation. For all graphs in this figure bars represent the mean values and standard error mean. Two-tailed unpaired *t* tests were used to evaluate significance. ****$P < 0.0001$; ***$P < 0.001$; **$P < 0.01$ and *$P < 0.1$.

 © The Author(s)
